# Analysis of Multivariate Disease Classification Data in the Presence of Partially Missing Disease Traits

**DOI:** 10.4172/2155-6180.1000197

**Published:** 2014-05-13

**Authors:** Jingang Miao, Samiran Sinha, Suojin Wang, W Ryan Diver, Susan M Gapstur

**Affiliations:** 1 Department of Statistics, Texas A&M University, College Station, TX 77843, USA; 2 Epidemiology Research Program, American Cancer Society, Inc. Atlanta, GA 30303, USA

**Keywords:** Dimension reduction, Disease classification, Estimating equations, Etiologic heterogeneity, Missing disease trait, Polytomous logistic model

## Abstract

In modern cancer epidemiology, diseases are classified based on pathologic and molecular traits, and different combinations of these traits give rise to many disease subtypes. The effect of predictor variables can be measured by fitting a polytomous logistic model to such data. The differences (heterogeneity) among the relative risk parameters associated with subtypes are of great interest to better understand disease etiology. Due to the heterogeneity of the relative risk parameters, when a risk factor is changed, the prevalence of one subtype may change more than that of another subtype does. Estimation of the heterogeneity parameters is difficult when disease trait information is only partially observed and the number of disease subtypes is large. We consider a robust semiparametric approach based on the pseudo-conditional likelihood for estimating these heterogeneity parameters. Through simulation studies, we compare the robustness and efficiency of our approach with that of the maximum likelihood approach. The method is then applied to analyze the associations of weight gain with risk of breast cancer subtypes using data from the American Cancer Society Cancer Prevention Study II Nutrition Cohort.

## Introduction

While disease trait information has been used in understanding survival of patients, relatively less research has been done on incorporating disease trait information into etiologic investigations. In this paper, we propose a new pseudo-conditional likelihood approach that can handle partially missing disease traits and use it to analyze data from the American Cancer Society’s Cancer Prevention Study (CPS) II Nutrition Cohort [1]. The goal of the data analysis is to investigate whether the association between weight gain and risk of breast cancer varies among different disease trait subtypes in women not using postmenopausal hormones, adjusting for important risk factors. If the association of a predictor variable varies across the subtypes, we examined how much of this variation is due to each of the disease traits. Understanding etiologic heterogeneity” of a risk factor sheds light on the pathogenesis of disease [2]. In the CPS-II Nutrition Cohort, there are 5 tumor characteristics, including stage (2 levels), histology (3 levels), estrogen receptor (2 levels), progesterone receptor (2 levels), and grade (3 levels), leading to 72 (i.e., 2×3×2×2×3) different disease subtypes.

To examine the effect of risk factors on different disease subtypes, we consider the polytomous logistic regression, which is commonly used for handling multinomial data [3–5]. There are two variants of the model: one for nominal and one for ordinal scale outcomes [6], and this paper focuses on modeling nominal outcomes. Hence, for each disease subtype, we have a set of disease-predictor association/regression parameters and a set of nuisance intercept parameters. The etiologic heterogeneity will be measured via differences among the regression parameters across subtypes. The number of regression parameters is large due to several disease characteristics (traits) while each trait has multiple levels. In this context, a second-stage model was proposed to reduce the dimension of the heterogeneity parameters when all disease traits are observed [7]. In the CPS-II Nutrition Cohort data, the missingness percentages for the five traits are 23.2%, 21.2%, 0.0%, 30.0%, and 33.6%, respectively. In particular, among the cases, approximately 45.5% had at least one missing trait.

While estimation of the heterogeneity parameters was considered in the Cox regression model in the presence of partially missing disease traits [8], the same issue has not been considered before in the context of polytomous logistic model, which will be considered in this paper. We propose to estimate the heterogeneity parameters using a pseudo-conditional likelihood. We would like to point out the distinction between Chatterjee [7] and our approach. Here we adopt the the secondstage model in a polytomous logistic regression setup in the presence of partially missing disease traits and develop a robust method of inference. In particular, Chatterjee [7] did not consider the missing data issue. As a result, his pseudo-conditional likelihood function was free of the nuisance intercept parameters. In contrast, we deal with partially missing disease trait data, and consequently our pseudo-conditional likelihood involves the nuisance intercept as well as the main log-odds ratio parameters. For estimating these nuisance parameters, we use a different type of pseudo-conditional likelihood. For handling the large dimension of the nuisance parameters, we adopt another second-stage model, and estimate them from another objective function. The idea of using two objective functions, one for the main parameters of interest and the other for the nuisance parameters, was inspired by Goetghebeur and Ryan [9]. Consequently the related theory is not a straightforward extension of the theory presented in [7].

Alternative to the proposed approach, one could consider a maximum likelihood based inference for the heterogeneity parameters using the full likelihood of the data. However, misspecification of the model for the intercepts will have less bearing on our inference than on the full likelihood based approach. Simulation studies clearly indicate this robustness property of our approach. Our inference is based on an artificially constructed pseudo-conditional likelihood function. To show its validity, we derive the large sample properties of the resulting estimator.

A brief outline of the remainder of the article is as follows. Section 2 contains the model and assumptions. In Section 3, we describe the proposed estimation methodology. The results of some simulation studies are described in Section 4. As an illustration, our method is applied to analyze the CPS-II Nutrition Cohort data in Section 5. Some concluding remarks are given in Section 6.

The Appendix contains the general methodology, the asymptotic properties, and the details of the simulation designs.

## Model and Notation

For each subject in a cohort of n subjects, when no missingness occurs we observe (*D*,*Y*,*X*), where D takes on one or zero according to whether the subject is diagnosed with the disease or not during the follow-up period. For the sake of simplicity and easy understanding, we shall consider only two disease traits (i.e., *K*=2) and assume that *X* is a scalar covariate (i.e., *P*=1) in Sections 2 and 3. The general case of K ≥ 2 and P ≥ 1 is described in Appendix A. Thus, *Y*=(*Y*_1_,*Y*_2_)*^T^* carries information on 2 disease traits. For a disease-free subject, we have *D*=0 and *Y*=(0,0)*^T^*. If the *k*-th trait has *M_k_* levels, then there are a total of *M*=*M*_1_×*M*_2_ disease subtypes. Our model is

(1) $$\begin{array}{l} p_{i,(y_{1},y_{2})}\equiv \text{pr}(D_{i}=1,Y_{i}=(y_{1},y_{2})|X_{i})=\frac{\exp(\alpha_{\left(y_{1},y_{2}\right)}+\beta_{\left(y_{1},y_{2}\right)}X_{i})}{1+\sum_{\left(y_{1},y_{2}\right)}\exp(\alpha_{\left(y_{1},y_{2}\right)}+\beta_{\left(y_{1},y_{2}\right)}X_{i})},\\ \text{pr}(D_{i}=0|X_{i})=\frac{1}{1+\sum_{\left(y_{1},y_{2}\right)}\exp(\alpha_{\left(y_{1},y_{2}\right)}+\beta_{\left(y_{1},y_{2}\right)}X_{i})}, \end{array}$$

for *i*=1,…,*n*, where *β*_(*y*_1_,*y*_2_)_ denotes the log-odds ratio parameter of the disease subtype (*y*_1_,*y*_2_) for the covariate, *α*_(*y*_1_,*y*_2_)_ denotes the nuisance intercept parameter, and Σ_(*y*_1_,*y*_2_)_ means summing over all M subtypes of the disease.

For a scalar continuous covariate scenario, there are *M* main regression (log-odds ratio) parameters of interest along with *M* intercept parameters, which are not the main interest here. Etiologic heterogeneity is measured via the differences among the regression parameters for a given covariate, and our focus is on estimation of the heterogeneity parameters.

### Second-stage model

To measure heterogeneity and reduce the dimension of subtype-specific regression parameters, following Chatterjee [7] we use the following second-stage model for the log-odds ratio parameters in model (1):

(2) $$\beta_{\left(y_{1},y_{2}\right)}=\theta^{\left(0\right)}+\theta_{1(y_{1})}^{\left(1\right)}+\theta_{2(y_{2})}^{\left(1\right)}+\theta_{12(y_{1},y_{2})}^{\left(2\right)},$$

where θ^(0)^ is the regression coefficient corresponding to the reference subtype of the disease, and the first-order and second-order parameter contrasts are respectively represented by $\theta_{k(y_{k})}^{\left(1\right)}$, *k* = 1,2, and $\theta_{12(y_{1},y_{2})}^{\left(2\right)}$. By assuming certain contrasts to be zero, we can reduce the number of parameters. In addition, these assumptions can be tested. Assuming the second- and higher-order contrasts are equal to zero, which we call a second-stage additive model, $\theta_{1(y)}^{\left(1\right)}-\theta_{1(y^{*})}^{\left(1\right)}$ tells us the degree of etiologic heterogeneity with respect to the first trait, regardless of the levels of other traits. For identifiability, we set $\theta_{1(1)}^{\left(1\right)}=\theta_{2(1)}^{\left(1\right)}=0$ *and* $\theta_{12(1,y_{2})}^{\left(2\right)}=\theta_{12(y_{1},1)}^{\left(2\right)}=0$. More elaborately, the heterogeneity of the log-odds ratio parameters due to the first trait can be measured via the contrasts $\theta_{1(2)}^{\left(1\right)},\ldots ,\theta_{1(M_{1})}^{\left(1\right)}$.

By assuming the second-order contrast parameters to be zero [7], we reduce the dimension of regression parameters from *M*_1_×*M*_2_ to 1+ *M*_1_−1+*M*_2_−1=*M*_1_+*M*_2_−1. In addition, in this case, the first-order contrast parameters directly measure etiologic heterogeneity. Usually the etiologic heterogeneity is measured via differences among the log-odds ratio parameters [10,11]. Chatterjee [7] first introduced the idea to express the log-odds ratio parameters in terms of different order contrast parameters, and this new idea has not been explored much. Importantly, the assumption regarding the contrast parameters are testable, provided data contain enough information regarding those parameters.

To simplify the notation in the second-stage model, we use a design matrix 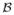 to relate the coefficient *β* that contains all the *β*_(*y*_1_,*y*_2_)_ parameters of the unstructured polytomous model to the parameters *θ* of the log-linear model (2) as *β*= 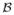
*θ*. In particular, $\beta_{\left(y_{1},y_{2}\right)}=B_{\left(y_{1},y_{2}\right)}^{T}\theta$, where $B_{\left(y_{1},y_{2}\right)}^{T}$ denotes the row of 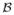 corresponding to disease subtype (*y*_1_,*y*_2_). Also, using a second-stage model we can write α= 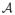
ξ, where α is a length-*M* vector of all *α*_(*y*_1_,*y*_2_)_ parameters. We use ξ to denote the second-stage parameters for the nuisance parameters. For clarity, we write $\alpha_{\left(y_{1},y_{2}\right)}=A_{\left(y_{1},y_{2}\right)}^{T}\xi$, where $A_{\left(y_{1},y_{2}\right)}^{T}$ denotes the row of 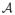 that corresponds to disease subtype (*y*_1_,*y*_2_).

Note that the use of the second-stage model for the regression parameters is not just for dimension reduction. More importantly, these second-stage model parameters are our main interest. As mentioned previously, these parameters directly measure the heterogeneity in the log-odds ratio parameters due to each of the disease trait. For the purpose of dimension reduction we set second and higher-order contrasts to be zero. However, this is not the only way of reducing dimension. For instance, one may keep all the second-stage model parameters, and then adopt the LASSO technique [12] to choose the important second-stage model parameters.

### Missingness mechanism

We introduce non-missing value indicator variables, *R_i_*=(*R_i_*_1_,*R_i_*_2_)*^T^*, where *R_ik_=*1 (k=1,2) if the *k*-th trait is observed for diseased subject *i* and 0 otherwise. Since for a non-diseased subject there is no relevance of disease traits, for all non-diseased subjects we set *R*=(1, 1)*^T^* for convenience. Note that there are at most 2^2^ types of missing data patterns: (0, 0), (0, 1), (1, 0), and (1, 1). For example, (1, 0) represents the case when the first trait is observed but not the second one. We assume that the probability of observing missingness pattern *r*, pr(*R*=*r*|*Y,X*)=*π*(*r,X*), does not depend on the disease traits. However, we not only allow the missingness probabilities to depend on X (a case of missing at random, MAR, [13,14]) but also allow the missingness indicators of different traits, R_1_ and R_2_, to be dependent on each other.

We introduce some additional notations to be used in the next sections. For the *i*-th subject, whose missing data pattern is *r*, we partition its vector of disease traits into the observed traits $y_{i}^{o_{r}}$ and the missing traits $y_{i}^{m_{r}}$. Similarly, we will use $\sum_{y_{i}^{m_{r}}}$ to sum over all the possible values of $y_{i}^{m_{r}}$. For example, if *Y*_1_=*y*_1_ but *Y*_2_ is missing, then *r=*(1, 0), *y*^*o*_*r*_^ = *y*_1_, *y*^*m*_*r*_^ = *Y*_2_, whose value is missing, and Σ_*y*^*m*_*r*_^_ means summing over all the terms corresponding to (*Y*_1_=*y*_1_, *Y*_2_=1), (*Y*_1_=*y*_1_, *Y*_2_=2),…,( *Y*_1_=*y*_1_, *Y*_2_=*M*_2_). When both traits are observed, Σ_*y*^*m*_*r*_^_ just uses the term corresponding to (*Y*_1_=*y*_1_, *Y*_2_=*y*_2_).

## Estimation Methodology

### Maximum likelihood method in the context of missing data

To estimate θ, one can use the maximum likelihood estimator (MLE), which is obtained by maximizing the full likelihood

$$L=\prod_{i=1}^{n}[{\left\{\frac{1}{1+\sum_{\left(y_{1},y_{2}\right)}\exp({A^{T}}_{\left(y_{1},y_{2}\right)}\xi +X_{i}{B^{T}}_{\left(y_{1},y_{2}\right)}\theta )}\right\}}^{1-D_{i}}\times \prod_{r}{\left\{\frac{\sum_{y_{i}^{m_{r}}}\exp\left(A_{\left(y_{i}^{o_{r}},y_{i}^{m_{r}}\right)}^{T}\xi +X_{i}B_{\left(y_{i}^{o_{r}},y_{i}^{m_{r}}\right)}^{T}\theta \right)}{1+\sum_{\left(y_{1},y_{2}\right)}\exp\left(A_{\left(y_{i},y_{2}\right)}^{T}\xi +X_{i}B_{\left(y_{1},y_{2}\right)}^{T}\theta \right)}\right\}}^{I(R_{i}=r)D_{i}}].$$

The resulting score functions for θ and ξ can be compactly written as

$$\begin{array}{l} S_{\theta }\equiv \frac{\partial \log(L)}{\partial \theta }=\sum_{i=1}^{n}\left\{D_{i}X_{i}\sum_{r}I\left(R_{i}=r\right)\sum_{y_{i}^{m_{r}}}B_{\left(y_{i}^{o_{r}},y_{i}^{m_{r}}\right)}\omega_{\left(y_{i}^{o_{r}},y_{i}^{m_{r}},X_{i}\right)}-X_{i}\sum_{\left(y_{1},y_{2}\right)}B_{\left(y_{1},y_{2}\right)}p_{i,(y_{1},y_{2})}\right\},\\ S_{\xi }\equiv \frac{\partial \log(L)}{\partial \xi }=\sum_{i=1}^{n}\left\{D_{i}\sum_{r}I\left(R_{i}=r\right)\sum_{y_{i}^{m_{r}}}A_{\left(y_{i}^{o_{r}},y_{i}^{m_{r}}\right)}\omega_{\left(y_{i}^{o_{r}},y_{i}^{m_{r}},X_{i}\right)}-\sum_{\left(y_{1},y_{2}\right)}A_{\left(y_{1},y_{2}\right)}p_{i,(y_{1},y_{2})}\right\}, \end{array}$$

where

$$\begin{array}{l} \omega_{\left(y_{i}^{o_{r}},y_{i}^{m_{r}},X_{i}\right)}=\frac{\exp(A_{\left(y_{i}^{o_{r}},y_{i}^{m_{r}}\right)}^{T}\xi +X_{i}B_{\left(y_{i}^{o_{r}},y_{i}^{m_{r}}\right)}^{T}\theta )}{\sum_{y_{i}^{m_{r}}}\exp(A_{\left(y_{i}^{o_{r}},y_{i}^{m_{r}}\right)}^{T}\xi +X_{i}B_{\left(y_{i}^{o_{r}},y_{i}^{m_{r}}\right)}^{T}\theta )},\\ p_{i,(y_{1},y_{2})}=\frac{\exp(A_{\left(y_{1},y_{2}\right)}^{T}\xi +X_{i}B_{\left(y_{1},y_{2}\right)}^{T}\theta )}{1+\sum_{\left(y_{1},y_{2}\right)}\exp(A_{\left(y_{1},y_{2}\right)}^{T}\xi +X_{i}B_{\left(y_{1},y_{2}\right)}^{T}\theta )}. \end{array}$$

If the model assumptions (see Appendix B) hold, then under standard regularity conditions given in Theorem 5.41 of [15], the *MLE η̃* = (*θ̃^T^*, *ξ̃^T^*)*^T^* asymptotically follows a normal distribution with mean *η* = (*θ^T^*, *ξ^T^*)*^T^*, and the asymptotic variance can be consistently estimated by {−(∂*S_η_*/∂*η^T^*)^−1^}*_η_*_=_*_η̃_*, where $S_{\eta }={\left(S_{\theta }^{T},S_{\xi }^{T}\right)}^{T}$,

$$\begin{array}{l} \frac{\partial S_{\theta }}{\partial \theta^{T}}=\sum_{i=1}^{n}X_{1}^{2}[D_{i}\sum_{r}I(R_{i}=r)\sum_{y_{i}^{m_{r}}}B_{\left(y_{i}^{o_{r}},y_{i}^{m_{r}}\right)}\omega_{\left(y_{i}^{o_{r}},y_{i}^{m_{r}},X_{i}\right)}\{B_{\left(y_{i}^{o_{r}},y_{i}^{m_{r}}\right)}^{T}-\sum_{y_{i}^{m_{r}}}B_{\left(y_{i}^{o_{r}},y_{r}^{m_{r}}\right)}^{T}\omega_{\left(y_{i}^{o_{r}},y_{i}^{m_{r}},X_{i}\right)}\}\\ -\sum_{\left(y_{1},y_{2}\right)}B_{\left(y_{1},y_{2}\right)}p_{i,(y_{1},y_{2})}\left\{B_{\left(y_{1},y_{2}\right)}^{T}-\sum_{\left(y_{1},y_{2}\right)}B_{\left(y_{1},y_{2}\right)}^{T}p_{i,(y_{1},y_{2})}\right\}],\\ \frac{\partial S_{\xi }}{\partial \theta^{T}}=\frac{\partial S_{\theta }}{\partial \xi^{T}}=\sum_{i=1}^{n}[X_{i}[D_{i}\sum_{r}I(R_{i}=r)\sum_{y_{i}^{m_{r}}}A_{\left(y_{i}^{o_{r}},y_{i}^{m_{r}}\right)}\omega_{\left(y_{i}^{o_{r}},y_{i}^{m_{r}},X_{i}\right)}\{B_{\left(y_{i}^{o_{r}},y_{i}^{m_{r}}\right)}^{T}-\sum_{y_{i}^{m_{r}}}B_{\left(y_{i}^{o_{r}},y_{r}^{m_{r}}\right)}^{T}\omega_{\left(y_{i}^{o_{r}},y_{i}^{m_{r}}\right)}\}\\ -\sum_{\left(y_{1},y_{2}\right)}A_{\left(y_{1},y_{2}\right)}p_{i,(y_{1},y_{2})}\left\{B_{\left(y_{1},y_{2}\right)}^{T}-\sum_{\left(y_{1},y_{2}\right)}B_{\left(y_{1},y_{2}\right)}^{T}p_{i,(y_{1},y_{2})}\right\}],\\ \frac{\partial S_{\xi }}{\partial \xi^{T}}=\sum_{i=1}^{n}[[D_{i}\sum_{r}I(R_{i}=r)\sum_{y_{i}^{m_{r}}}A_{\left(y_{i}^{o_{r}},y_{i}^{m_{r}}\right)}\omega_{\left(y_{i}^{o_{r}},y_{i}^{m_{r}},X_{i}\right)}\{A_{\left(y_{i}^{o_{r}},y_{i}^{m_{r}}\right)}^{T}-\sum_{y_{i}^{m_{r}}}\omega_{\left(y_{i}^{o_{r}},y_{i}^{m_{r}},X_{i}\right)}A_{\left(y_{i}^{o_{r}},y_{r}^{m_{r}}\right)}^{T}\}\\ -\sum_{\left(y_{1},y_{2}\right)}A_{\left(y_{1},y_{2}\right)}p_{i,(y_{1},y_{2})}\left\{A_{\left(y_{1},y_{2}\right)}^{T}-\sum_{\left(y_{1},y_{2}\right)}A_{\left(y_{1},y_{2}\right)}^{T}p_{i,(y_{1},y_{2})}\right\}]. \end{array}$$

As evident from the above discussion, the inference of the heterogeneity parameters, *θ*, depends on the intercept parameters *α* and their model *α*= 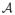
ξ.. Next we discuss an alternative inference for the heterogeneity parameters, which is more robust against the misspecification of the second-stage model for *α*.

### Pseudo-conditional likelihood in the context of missing data

In order to form pseudo-conditional likelihoods (PCL), for every subject with disease, we define a matched set 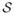 consisting of the subject itself and all subjects without the disease. Thus, if *D_i_*=1, then *S_i_* = {*i*}∪{*j: D_j_* =0}. If there are *n*_0_ controls, then the cardinality of 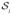 is (*n*_0_ + 1). We form the pseudo-conditional likelihood 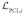 such that the *i*-th subject has a disease of subtype ( $y_{i}^{o_{r}},y_{i}^{m_{r}}$) given that there is only one subject with disease ( $y_{i}^{o_{r}},y_{i}^{m_{r}}$) in the set 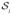:

$$\begin{array}{l} L_{\mathrm{PCL},i}=\prod_{r}{\left\{\sum_{y_{i}^{m_{r}}}\frac{\text{pr}(D_{i}=1,Y_{i}=(y_{i}^{o_{r}},y_{i}^{m_{r}})|X_{i})\prod_{j\in S_{i}\backslash \{i\}}\text{pr}(D_{j}=0|X_{j})}{\sum_{k\in S_{i}}\sum_{y_{i}^{m_{r}}}\text{pr}(D_{k}=1,Y_{k}=(y_{i}^{o_{r}},y_{i}^{m_{r}})|X_{k})\prod_{j\in S_{i}\backslash \{k\}}\text{pr}(D_{j}=0|X_{j})}\right\}}^{I(R_{i}=r)D_{i}}\\ =\prod_{r}{\left\{\frac{\sum_{y_{i}^{m_{r}}}\exp(A_{y_{i}^{o_{r}},y_{i}^{m_{r}}}^{T}\xi +X_{i}B_{\left(y_{i}^{o_{r}},y_{i}^{m_{r}}\right)}^{T}\theta )}{\sum_{j\in S_{i}}\sum_{y_{i}^{m_{r}}}\exp(A_{\left(y_{i}^{o_{r}},y_{i}^{m_{r}}\right)}^{T}\xi +X_{i}B_{\left(y_{i}^{o_{r}},y_{i}^{m_{r}}\right)}^{T}\theta )}\right\}}^{I(R_{i}=r)D_{i}}. \end{array}$$

Then the pseudo-conditional likelihood is defined as the product of 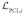 over *i*, i.e., $L_{\mathrm{PCL}}=\prod_{i=1}^{n}L_{\mathrm{PCL},i}$, and the estimating functions are defined as the derivatives of log( 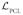) with respect to θ:

$$\begin{array}{l} S_{EE,\theta }\equiv \frac{\partial \log(L_{PCL})}{\partial \theta }=\sum_{i=1}^{n}D_{i}\sum_{r}I(R_{i}=r)\{X_{i}\sum_{y_{i}^{m_{r}}}B_{\left(y_{i}^{o_{r}},y_{i}^{m_{r}}\right)}\omega_{\left(y_{i}^{o_{r}},y_{i}^{m_{r}},X_{i}\right)}\\ \frac{\sum_{j\in S_{i}}X_{j}\sum_{y_{i}^{m_{r}}}\exp(A_{\left(y_{i}^{o_{r}},y_{i}^{m_{r}}\right)}^{T}\xi +X_{j}B_{\left(y_{i}^{o_{r}},y_{i}^{m_{r}}\right)}^{T}\theta )B_{\left(y_{i}^{o_{r}},y_{i}^{m_{r}}\right)})}{\sum_{j\in S_{i}}\sum_{y_{i}^{m_{r}}}\exp(A_{\left(y_{i}^{o_{r}},y_{i}^{m_{r}}\right)}^{T}\xi +X_{j}B_{\left(y_{i}^{o_{r}},y_{i}^{m_{r}}\right)}^{T}\theta )}\}=0. \end{array}$$

Note that 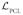 is free of ξ(or α*_y_*) if there are no missing disease traits for any of the diseased subjects. Therefore, 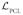 contains somewhat limited information regarding ξ. Hence, we shall estimate ξ from another set of estimating equations. Goetghebeur and Ryan [9] first introduced two different sets of estimating equations in the context of missing causes of failure in the competing risk model. Here, to estimate ξ we consider another pseudo-conditional likelihood 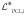 such that the *i*-th subject has a disease of subtype ( $y_{i}^{o_{r}},y_{i}^{m_{r}}$) given that there is only one diseased subject in 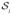 without specifying the observed disease subtype. It is given as

$$L_{\mathrm{PCL},i}=\prod_{r}{\left\{\sum_{y_{i}^{m_{r}}}\frac{\text{pr}(D_{i}=1,Y_{i}=(y_{i}^{o_{r}},y_{i}^{m_{r}})|X_{i})\prod_{j\in S_{i}\backslash \{i\}}\text{pr}(D_{j}=0|X_{j})}{\sum_{k\in S_{i}}\sum_{y_{i}^{m_{r}}}\text{pr}(D_{k}=1,Y_{k}=(y_{i}^{o_{r}},y_{i}^{m_{r}})|X_{k})\prod_{j\in S_{i}\backslash \{k\}}\text{pr}(D_{j}=0|X_{j})}\right\}}^{I(R_{i}=r)D_{i}}$$

Hence, by defining $L_{\text{PCL}}^{*}=\prod_{i=1}^{n}L_{\text{PCL},i}^{*}$, the estimating equations for ξ are

$$S_{EE,\xi }\equiv \frac{\partial \log(L_{\mathrm{PCL}}^{*})}{\partial \xi }=\sum_{i=1}^{n}D_{i}\{\sum_{r}I(R_{i}=r)\sum_{y_{i}^{m_{r}}}A_{y_{i}^{o_{r}},y_{i}^{m_{r}}}\omega_{y_{i}^{o_{r}},y_{i}^{m_{r}}}-\frac{\sum_{j\in S_{i}}\sum_{\left(y_{1},y_{2}\right)}\exp(A_{\left(y_{1},y_{2}\right)}^{T}\xi +X_{j}B_{\left(y_{1},y_{2}\right)}^{T}\theta )A_{\left(y_{1},y_{2}\right)}^{T}}{\sum_{j\in S_{i}}\sum_{\left(y_{1},y_{2}\right)}\exp(A_{\left(y_{1},y_{2}\right)}^{T}\xi +X_{j}B_{\left(y_{1},y_{2}\right)}^{T}\theta )}\}=0.$$

We estimate θ and ξ by solving *S*_EE,θ_=0 and *S*_EE,ξ_=0 simultaneously. Denote the resulting estimates as *η̂* = (*θ̂^T^*, *ξ̂^T^*)*^T^*. The estimating equations are asymptotically unbiased, as is shown in Appendix B. The asymptotic distribution of the estimators is multivariate normal with the asymptotic covariance of *η̂* consistently estimated by a sandwich estimator. The middle component of the sandwich estimator is obtained via a linearization technique applied to the estimating equations. The left and right multipliers of the sandwich estimator are the derivative of the estimating equations with respect to the parameters. See Appendix B for the general case.

## Simulation Studies

### Simulation design

One of the main goals of this numerical investigation was to show how robust our method is towards a misspecification of the intercept model in the presence of partially missing disease traits. We simulated cohort data of size n=5,000 by simulating (*X*,*Y*,*D*). The scalar covariate *X* was simulated from the Normal(0,1) distribution. We considered two scenarios each with 3 traits. First with 8=(2×2×2) disease subtypes, and second with 30 (=2×3×5) disease subtypes. For each scenario we considered a correctly specified (denoted by a) second-stage model and a misspecified one (denoted by b) for the intercepts. We created missing values in each trait where missingness probabilities depended on *X*. Two mechanisms were used: *M*_1_) the missingness probabilities were dependent on *X* but the missingness of different traits was independent; and *M*_2_) the missingness probabilities were dependent on *X* and the missingness of different traits was dependent. Overall disease probability lies between 6% and 9%.

For scenario 1, we considered three disease characteristics each with two levels, resulting in 2×2×2=8 disease subtypes. Assuming that the second- and higher-order contrasts for the relative risk parameters are negligible, we write

$$\beta =B\theta ,\beta =\left[\begin{array}{c} \beta_{\left(1,1,1\right)}\\ \beta_{\left(1,1,2\right)}\\ \beta_{\left(1,2,1\right)}\\ \beta_{\left(1,2,2\right)}\\ \beta_{\left(2,1,1\right)}\\ \beta_{\left(2,1,2\right)}\\ \beta_{\left(2,2,1\right)}\\ \beta_{\left(2,2,2\right)} \end{array}\right],B=\left[\begin{array}{cccc} 1 & 0 & 0 & 0\\ 1 & 0 & 0 & 1\\ 1 & 0 & 1 & 0\\ 1 & 0 & 1 & 1\\ 1 & 1 & 0 & 0\\ 1 & 1 & 0 & 1\\ 1 & 1 & 1 & 0\\ 1 & 1 & 1 & 1 \end{array}\right],\theta ={\left(\theta^{\left(0\right)},\theta_{1(2)}^{\left(1\right)},\theta_{2(2)}^{\left(1\right)},\theta_{3(2)}^{\left(1\right)}\right)}^{T},$$

and we chose θ=(0.35, 0.15, 0, 0.5)^T^. Thus the disease subtypes were generated using the model pr(*Y*=(*y*_1_,*y*_2_,*y*_3_)|*X*) = exp(*α*_(*y*_1_,*y*_2_,*y*_3_)_+*β*_(*y*_1_,*y*_2_,*y*_3_)_*X*){1+Σ_(*y*_1_,*y*_2_,*y*_3_)_ exp(*α*_(*y*_1_,*y*_2_,*y*_3_)_+*β*_(*y*_1_,*y*_2_,*y*_3_)_*X*)}^−1^.We chose *α*_(*y*_1_,*y*_2_,*y*_3_)_ to follow the same model as *β*_(*y*_1_,*y*_2_,*y*_3_)_ with 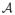
= 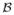 and ξ=(−5,0,0,0)*^T^* (scenario1a). In addition, to study the robustness of the approach against the misspecification of the model for the intercepts (scenario 1b), we used α=(−5.193,−4.477,−5.297,−5.033,−5.170,−5.160,−4.340,−5.330)*^T^* by adding vector (−5,−5,−5,−5,−5,−5,−5,−5)*^T^* in the column space of 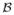, which is the correctly specified part, to vector (−0.193,0.523,−0.297,−0.033,−0.170,−0.160, 0.660,−0.330)*^T^* perpendicular to the column space, which is the misspecified part.

Finally, we created missing values in the diseases traits using two mechanisms. For *M*_1_, the missing probabilities for each of the traits were allowed to depend on *X* through the logistic function exp(−1.5+0.5*X*) {1+exp(−1.5+0.5*X*)}^−1^, resulting in missingness probabilities of around 0.2 for each disease trait. For *M*_2_, 3 traits had 2^3^=8 possible missingness patterns. For each case subject these patterns were generated from a multinomial distribution with the following probabilities pr{R=(1,0,0)|*X*}=d^−1^exp(γ_1_+0.5*X*); pr{R=(0,1,0)|X}=d^−1^exp(γ_2_+0.5*X*); pr{R=(1,1,0)|*X*}=d^−1^exp(γ_3_+0.5*X*); pr{R=(0,0,1)|*X*}=d^−1^exp(γ_4_+0.5*X*); pr{R=(1,0,1)|*X*}=d^−1^exp(γ_5_+0.5*X*); pr{R=(0,1,1)|*X*}=d^−1^exp(γ_6_+0.5*X*); pr{R=(1,1,1)|*X*}=d^−1^exp(γ_7_+0.5*X*), where $d=1+\sum_{i=1}^{7}\exp(\gamma_{i}+0.5X)$ and γ_1_,…,γ_7_ were chosen so that marginally each trait had about 20% missing values.

For scenario 2, we considered three disease traits with numbers of levels 2, 3, and 5, resulting in 2×3×5=30 disease subtypes. With the corresponding 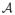
= 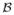 defined by the second-stage additive model, we took θ=(0.35,0.15,0,0.5,0.35,0.15,0,0.5)*^T^* and ξ=(−5,0,0,0,0,0,0,0)*^T^* (scenario 2a). For scenario 2b, we chose α the same way as in scenario 1b.

Finally, we created missing values in the disease traits. For mechanism one, the missingness probabilities were allowed to depend on *X* through the logistic function exp(γ*_k_*+0.5*X*){1+exp((γ*_k_*+0.5*X*)}^−1^, where γ*_k_* was chosen to be (−1.5,−1.5,−0.85)*^T^*, resulting in missing probabilities of around 0.2, 0.2, and 0.3 for the three disease traits, respectively. For mechanism two, we allowed the missingness probabilities to depend on each other in a similar pattern as in scenario 1.

### Method of analysis

Each of the simulated datasets was analyzed by the maximum likelihood approach (MLE) and by the pseudo-conditional likelihood method (PCL). Furthermore, we analyzed the data considering only the subjects without any missing disease traits using the maximum likelihood approach, and we refer to it as the complete-case maximum likelihood estimator (CMLE). In all these analyses, we adopted the second-stage additive models for the regression and intercept parameters, β= 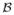
θ and α= 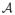
ξ. We present mean, median, median absolute deviation (MAD), empirical standard errors (Emp. SE), estimated standard errors (Est. SE), 95% coverage probabilities, and root mean square errors (RMSE) of all the methods based on 2,000 replications. To assess asymptotic bias, we present $B.\text{score}=\sqrt{2000}(\text{mean}\;\text{estimate}-\text{truth})/\text{Emp}.\text{SE}$.

## Results

To save space, in both scenarios we omit the results for missingness mechanism two, which are very similar to those for mechanism one. Also, we leave out results for the correctly specified intercept model case in scenario two. The conclusions that could be drawn from the results not presented were not different from those presented here. We would be happy to provide these omitted results upon request. The results for scenarios 1a (top panel of Table 1) indicate that when the intercept model is correctly specified: (1) all three methods are asymptotically unbiased; (2) the standard errors of the PCL method were slightly larger than that of the MLE method, but smaller than that of the CMLE method, which suggests that the PCL’s efficiency is close to that of the MLE method; (3) similar to the standard errors, the RMSEs of the PCL method were slightly larger than that of the MLE method, but smaller than that of the CMLE method; (4) the estimated standard errors of the PCL method were close to that of the empirical standard errors; and (5) all methods’ coverage probabilities were close to the nominal level (95%). The trend of the results remains the same for scenario 2a.

The results for scenarios 1b (bottom panel of Table 1) and 2b (Table 2) indicate that when the intercept model is misspecified: (1) the biases of both the MLE and the CMLE methods were prominent, but the biases of the PCL method were far less serious; (2) the comparisons of the three methods in terms of standard errors, RMSEs and estimated and empirical standard errors agreement were similar to those in the model with correctly specified model for the intercepts; and (3) the coverage probabilities of the MLE and the CMLE methods deviated from the nominal level, but the coverage probabilities of the PCL stayed close to the nominal level. Finally, the PCL method was almost as efficient as the MLE method in all scenarios. The bias of the CMLE method can be attributed to model misspecification of the model for the intercepts and ignoring the subjects with missing traits. However, the main source of bias in the MLE method is due to model misspecification.

Following a referee’s comment we conducted additional simulation to study the performance of the three methods in the presence of non-null second-order contrasts in the true data generating process. As in Scenario 1, we used 2×2×2=8 disease subtypes, the missingness probabilities were made depended on *X*, and the intercept model was misspecified. But in addition to the original θ=(0.35, 0.15, 0, 0.5)*^T^*, the true values of the second-order contrast parameters were taken as $\theta_{12(2,2)}^{\left(2\right)}=0,\theta_{13(2,2)}^{\left(2\right)}=-0.2,\theta_{23(2,2)}^{\left(2\right)}=0.2$. We call this Scenario 1c. For Scenario 1c, we first analyzed the simulated datasets assuming a second-stage additive model, meaning second- and higher-order contrast were set to zero. Then, we analyzed the datasets adopting a second-stage model keeping all first- and second-order contrasts parameters but setting third- and higher-order contrast parameters to zero. For the misspecified additive model (top panel of Table 3), the PCL method’s biases were much smaller than either MLE or CMLE for all but one parameters, and its RMSE’s were smaller than CMLE and sometimes smaller than MLE. With the second-order contrasts included in the model (bottom panel of Table 3) the PCL method also performed well with the smallest biases.

### Data Example

The CPS-II Nutrition Cohort is a prospective study of cancer incidence and mortality in 86,402 men and 97,786 women and has been described in detail elsewhere [1]. Briefly, the Nutrition Cohort is a subgroup of the approximately 1.2 million participants of the CPS-II Cohort, a prospective study of cancer mortality established by the American Cancer Society in 1982 [16]. Nutrition Cohort participants resided in 21 states with population-based cancer registries, were aged 50–74 years, and completed a 10-page confidential, self-administered mailed questionnaire at enrollment in 1992 or 1993.

Excluded from this analysis were Nutrition Cohort participants who were men (*n*=86,402); women who were using hormone replacement therapy (*n*=33,407), not post-menopausal (*n=*3,514), lost to follow-up (i.e., alive at the first follow-up questionnaire in 1997 but did not return the 1997 or any subsequent follow-up questionnaires) (*n*=2,178), reported a personal history of cancer other than non-melanoma skin cancer in 1992 (*n*=9,520), reported a diagnosis of breast cancer on the first survey that could not be verified through medical or cancer registry records or an in situ breast cancer (*n*=174), or the subjects with missing values in any of the predictor variables or whose weight gain was more than 100 lbs (*n*=7,979). Included in the analysis were 41,014 women. There were 1,555 incident cases of breast cancer (International Classification of Disease for Oncology, Second and Third Editions site code C50) that occurred between the date of the baseline questionnaire and June 30, 2007.

The risk factor of interest in the analysis was total weight change since age 18 to 1992 (WG) as it has been shown to be related to risk of breast cancer in previous studies (e.g., [8], [17], and [18]). WG was transformed to be between 0 and 1 for numerical stability. Using (*y*_1_,…,*y*_5_) to represent levels of the five traits, stage (2 levels), histology (3 levels), estrogen receptor (2 levels), progesterone receptor (2 levels), and grade (3 levels), we can write the polytomous logistic model and the corresponding second-stage additive model as

$$\begin{array}{l} \text{pr}(D_{i}=1,Y_{i}=(y_{1},\ldots ,y_{5})|X_{i})=\frac{\exp(\alpha_{\left(y_{1},\ldots ,y_{5}\right)}+\beta_{\left(y_{1},\ldots ,y_{5}\right)}X_{i})}{1+\sum_{y}\exp(\alpha_{\left(y_{1},\ldots ,y_{5}\right)}+\beta_{\left(y_{1},\ldots ,y_{5}\right)}X_{i})},\\ \text{pr}(D_{i}=0,|X_{i})=\frac{1}{1+\sum_{\left(y_{1},\ldots ,y_{5}\right)}\exp(\alpha_{\left(y_{1},\ldots ,y_{5}\right)}+\beta_{\left(y_{1},\ldots ,y_{5}\right)}X_{i})},\\ \beta_{\left(y_{1},\ldots ,y_{5}\right)}=\theta^{\left(1\right)}+{\theta^{\left(1\right)}}_{1(y_{1})}+{\theta^{\left(1\right)}}_{2(y_{2})}+{\theta^{\left(1\right)}}_{3(y_{3})}+{\theta^{\left(1\right)}}_{4(y_{4})}+{\theta^{\left(1\right)}}_{5(y_{5})}, \end{array}$$

for *i*=1,…,*n*. Contingency tables for the disease configurations can be found in Table 4. We used the second-stage additive models for both the intercepts and regression (log-odds ratio) parameters for all three methods. For the MLE and PCL methods we used all 1,555 cases while for the CMLE approach we used 848 cases whose disease traits information was complete.

The results are presented in Table 5. Since the PCL approach is more robust towards misspecification of the intercept model, we interpret the corresponding results here. Under the PCL method, we conclude that (1) the estimate of θ^(0)^ due to weight gain is positive and statistically significant at the 5% level. The odds ratio for the incidence of breast cancer with well differentiated grade, localized stage, histology ductal, ER status positive and PR status positive for the 3rd quartile (45 lbs, re-scaled to be 0.476) of weight gain versus 1st quartile of weight gain (15 lbs, re-scaled to be 0.190) is 1.356 (exp{(0.476 – 0.190) × 1:066}, 95% confidence interval (CI): 1.164–1.580); (2) the PCL method produced statistically significant estimates of $\theta_{2(2)}^{\left(1\right)},\theta_{3(2)}^{\left(1\right)},\theta_{4(2)}^{\left(1\right)}$, and $\theta_{5(2)}^{\left(1\right)}$ for the covariate weight gain, which can be interpreted as follows. For a women who gained 45 pounds versus one who gained 15 pounds, the odds ratio of the disease with distant tumor is 1.260 (95% CI: 1.089–1.459) times the odds ratio of the disease with localized tumor, keeping all other traits fixed; the odds ratio of the disease with lobular histology is 0.822 (95% CI: 0.694–0.974) times the odds ratio of the disease with ductal histology, keeping all other traits fixed; the odds ratio of the disease with ER− status is 1.287 (95% CI: 1.006–1.646) times the odds ratio of the disease with ER+ status, keeping all other traits fixed; the odds ratio of the disease with PR–status is 0.705 (95% CI: 0.577–0.862) times the odds ratio of the disease with PR+ status, keeping all other traits fixed.

Following a referee’s suggestion, we conducted a model assessment for the data example. There are 72 log-odds ratio parameters, and as will be discussed in the last paragraph of this section, not all of these parameters are estimable. Now, we consider a second-stage model where all third- and higher-order contrast are zero. In this setup we test H_0_: all second-order contrasts are zero against H_a_: at least one of the second-order contrasts is non-zero. For this purpose we fit the model with all first- and second-order contrast parameters using the proposed PCL approach. The test statistic is *T* =(*Aθ̂*)*^T^* (*A*Σ *A^T^*)^−1^ *Aθ̂*, where A is a 19 × 27 matrix partitioned as *A*=(**0**_19×8_: ***I***_19_) with ***I***_19_ being an identity matrix of order 19, and Σ stands for the asymptotic variance covariance matrix for *θ̂*. Under *H*_0_, *T* approximately follows the $\chi_{19}^{2}$ distribution. The corresponding *p*-value was smaller than 0.001, indicating that some second-order contrast parameters significantly (at the 5% level) differ from zero. Please see Table 6 for the new analysis with the second-stage model containing all first- and second-order contrast parameters. Although complex due to the presence of some non-null second-order contrast parameters, the model parameters of Table 6 can be interpreted. For example, we interpret $\theta_{14(3,2)}^{\left(2\right)}$ as follows:

$$\exp\left\{\theta_{14(3,2)}^{\left(2\right)}\right\}=\frac{\left\{\text{pr}(G=\text{Poor},\text{ER}=-|X+1)/\text{pr}(G=\text{Well},\text{ER}=-|X+1)\}/\{\text{pr}(G=\text{Poor},\text{ER}=-|X)/\text{pr}(G=\text{Well},\text{ER}=-|X)\right\}}{\left\{\text{pr}(G=\text{Poor},\text{ER}=+|X+1)/\text{pr}(G=\text{Well},\text{ER}=+|X+1)\}/\{\text{pr}(G=\text{Poor},\text{ER}=+|X)/\text{pr}(G=\text{Well},\text{ER}=+|X)\right\}}$$

Where G stands for Grade. Here the numerator is the odds ratio for Grade being Poor vs. Well associated with one unit increase in weight gain when ER status is −, whereas the denominator is the same odds ratio when ER status is +. Here $\theta_{14(3,2)}^{\left(2\right)}$ is non-zero, so the odds ratio varies with the change of ER status. Also, due to estimation of more parameters, the standard errors of the estimators have substantially increased (please see the standard errors of the first-order contrast parameters in the PCL method in Table 5 vs. Table 6. This entire testing procedure demonstrates one of the good features of the proposed method that we can formally test our assumptions regarding the contrast parameters.

Prompted by a reviewer’s comment, here we discuss the issue of configurations with few or no subjects. There are no subjects in 23 out of the 72 possible disease subtypes. That means that a simple polytomous logistic model cannot be fit to this data with all 72 disease subtypes. The second-stage additive model, on the other hand, can enable us to make use of the cross classification structure and thus achieve sharing information across subtypes. The proposed method with second stage additive model still works when some subtypes have no cases observed. We require some cases for every level of each trait, which is easier to have than requiring cases for each subtype. Moreover, in the data example, our method works when the second-stage model contains all first- and second order contrast parameters. In fact, one may add more higher-order contrast parameters in the second-stage model, but these additional parameters may not be estimable from the data. For example, the third- and higher-order contrast parameters involving ER−, Grade Well, and Stage Distant are not estimable as the corresponding cell frequency is zero (the third panel of Table 4).

## Discussion

The two-stage model is an efficient and flexible way to measure heterogeneity of the odds ratios. It allows a sensible way to dimension reduction. For parameter estimation of the second-stage model, one can use the MLE, PCL, or the CMLE methods. Compared with the MLE method, our method reduces the effects of the intercepts on the estimation of the regression parameters, and thus it is more robust against the misspecification of the model for the intercepts.

When the model is correct, the PCL method is asymptotically unbiased. In addition, our simulations suggest (1) when the second-stage model for the intercepts is misspecified, our bias is usually smaller than that of either the MLE method or the CMLE method, and (2) with either correctly specified or misspecified model for the intercepts, our method can usually achieve efficiency that is very close to the MLE method.

Analysis of the Cancer Prevention Study (CPS)-II Nutrition Cohort data represents the first effort that the authors are aware of to simultaneously examine the effect of multiple covariates on the outcome. We hope that it not only is a demonstration of the method but also sheds light on the etiology of breast cancer.

## Figures and Tables

**Table 1:** Simulation results for the complete-case MLE, the MLE, and the pseudo-conditional likelihood method. Here MAD, Emp. SE, Est. SE, Bias, B. Score, RMSE, and CP denote median absolute deviation, empirical standard error, estimated standard error, bias, bias score, root mean squared error, and 95% coverage probability based on the Wald-type confidence intervals, respectively. The results were based on 2,000 replications. There were 2×2×2=8 disease subtypes. The missingness probabilities depended on the covariate.

Scenario 1a: Correctly Specified Model for Intercepts												
	Complete-case MLE				MLE				Pseudo-conditional Likelihood Method			
	θ^(0)^ =0.35	$\theta_{1(2)}^{\left(1\right)}=0.15$	$\theta_{2(2)}^{\left(1\right)}=0$	$\theta_{3(2)}^{\left(1\right)}=0.5$	θ^(0)^ =0.35	$\theta_{1(2)}^{\left(1\right)}=0.15$	$\theta_{2(2)}^{\left(1\right)}=0$	$\theta_{3(2)}^{\left(1\right)}=0.5$	θ^(0)^ =0.35	$\theta_{1(2)}^{\left(1\right)}=0.15$	$\theta_{2(2)}^{\left(1\right)}=0$	$\theta_{3(2)}^{\left(1\right)}=0.5$
Mean	0.357	0.149	0.000	0.497	0.354	0.147	0.001	0.498	0.351	0.148	0.002	0.500
Median	0.353	0.149	0.003	0.498	0.349	0.146	0.004	0.496	0.348	0.146	0.005	0.498
MAD	0:170	0.157	0.161	0.154	0.127	0.130	0.123	0.128	0.129	0.133	0.129	0.127
Emp. SE	0.161	0.152	0.153	0.158	0.126	0.128	0.125	0.126	0.129	0.132	0.128	0.128
Est. SE	0.163	0.153	0.152	0.159	0.129	0.125	0.124	0.129	0.131	0.128	0.128	0.133
Bias	0.007	−0.001	0.000	−0.003	0.004	−0.003	0.001	−0.002	0.001	−0.002	0.002	−0.000
B. Score	1.985	−0.189	0.046	−0.720	1.406	−0.904	0.455	−0.791	0.448	−0.550	0.693	−0.064
RMSE	0.161	0.152	0.153	0.158	0.127	0.128	0.125	0.126	0.129	0.132	0.128	0.128
CP	0.948	0.955	0.952	0.949	0.958	0.949	0.948	0.956	0.950	0.948	0.951	0.963

Scenario 1a: Correctly Specified Model for Intercepts												
	Complete-case MLE				MLE				Pseudo-conditional Likelihood Method			
	θ^(0)^ =0.35	$\theta_{1(2)}^{\left(1\right)}=0.15$	$\theta_{2(2)}^{\left(1\right)}=0$	$\theta_{3(2)}^{\left(1\right)}=0.5$	θ ^(0)^ =0.35	$\theta_{1(2)}^{\left(1\right)}=0.15$	$\theta_{2(2)}^{\left(1\right)}=0$	$\theta_{3(2)}^{\left(1\right)}=0.5$	θ ^(0)^ =0.35	$\theta_{1(2)}^{\left(1\right)}=0.15$	$\theta_{2(2)}^{\left(1\right)}=0$	$\theta_{3(2)}^{\left(1\right)}=0.5$
Mean	0.475	0.027	−0.077	0.456	0.476	0.023	−0.076	0.459	0.383	0.122	−0.017	0.482
Median	0.478	0.031	−0.077	0.451	0.476	0.022	−0.075	0.457	0.383	0.120	−0.016	0.479
MAD	0.147	0.146	0.155	0.147	0.117	0.125	0.124	0.122	0.133	0.130	0.134	0.124
Emp. SE	0.148	0.150	0.152	0.152	0.117	0.122	0.124	0.122	0.133	0.128	0.130	0.127
Est. SE	0.159	0.149	0.149	0.155	0.126	0.122	0.122	0.125	0.136	0.128	0.127	0.132
Bias	0.125	−0.123	−0.077	−0.044	0.126	−0.127	−0.076	−0.041	0.033	−0.028	−0.017	−0.018
B. Score	37.776	−36.571	−22.603	−12.880	48.513	−46.309	−27.410	−15.099	11.160	−9.918	−5.717	−6.248
RMSE	0.194	0.194	0.171	0.158	0.172	0.176	0.145	0.129	0.137	0.131	0.131	0.129
CP	0.898	0.860	0.916	0.942	0.858	0.812	0.898	0.942	0.954	0.946	0.946	0.954

**Table 2:** Simulation results for the complete-case MLE, the MLE, and the pseudo-conditional likelihood method. Here MAD, Emp. SE, Est. SE, Bias, B. Score, RMSE, and CP denote median absolute deviation, empirical standard error, estimated standard error, root mean squared error, bias, bias score, root mean squared error, and 95% coverage probability based on the Wald-type confidence intervals, respectively. The results were based on 2,000 runs. There were 2×3×5 = 30 disease subtypes. The model for the intercepts was misspecified. The missingness probabilities depended on the covariate. This is Scenario 2b.

	θ^(0)^=0.35	$\theta_{1(2)}^{\left(1\right)}=0.15$	$\theta_{2(2)}^{\left(1\right)}=0$	$\theta_{2(3)}^{\left(1\right)}=0.5$	$\theta_{3(2)}^{\left(1\right)}=0.35$	$\theta_{3(3)}^{\left(1\right)}=0.15$	$\theta_{3(4)}^{\left(1\right)}=0$	$\theta_{3(5)}^{\left(1\right)}=0.5$
Complete-case MLE								
Mean	0.475	0.154	0.004	0.425	0.251	0.017	−0.145	0.407
Median	0.473	0.154	0.004	0.426	0.252	0.014	−0.144	0.411
MAD	0.184	0.134	0.180	0.166	0.209	0.204	0.218	0.212
Emp. SE	0.185	0.131	0.178	0.164	0.214	0.217	0.221	0.207
Est. SE	0.190	0.134	0.182	0.166	0.209	0.217	0.221	0.204
Bias	0.125	0.004	0.004	−0.075	−0.099	−0.133	−0.145	−0.093
B. Score	30.220	1.249	1.066	−20.372	−20.787	−27.353	−29.367	−20.149
RMSE	0.223	0.131	0.178	0.180	0.236	0.254	0.265	0.227
CP	0.910	0.954	0.960	0.930	0.917	0.911	0.0009	0.920
MLE								
Mean	0.474	0.153	0.006	0.428	0.252	0.012	−0.148	0.402
Median	0.474	0.150	0.005	0.428	0.252	0.008	−0.148	0.402
MAD	0.139	0.102	0.133	0.120	0.171	0.172	0.185	0.170
Emp. SE	0.144	0.101	0.136	0.126	0.171	0.176	0.182	0.168
Est. SE	0.147	0.102	0.139	0.126	0.171	0.178	0.181	0.166
Bias	0.124	0.003	0.006	−0.072	−0.098	−0.138	−0.148	−0.098
B. Score	38.546	1.148	1.919	−25.553	−25.665	−35.047	−36.248	−26.070
RMSE	0.190	0.101	0.136	0.146	0.197	0.223	0.235	0.194
CP	0.881	0.959	0.954	0.910	0.909	0.886	0.864	0.902
Pseudo-conditional Likelihood Method								
Mean	0.381	0.160	0.001	0.476	0.327	0.119	−0.039	0.478
Median	0.379	0.156	−0.000	0.473	0.330	0.115	−0.039	0.479
MAD	0.156	0.108	0.138	0.131	0.177	0.173	0.190	0.178
Emp. SE	0.157	0.108	0.139	0.132	0.179	0.180	0.191	0.177
Est. SE	0.164	0.111	0.141	0.165	0.192	0.188	0.188	0.208
Bias	0.031	0.010	0.001	−0.024	−0.023	−0.031	−0.039	−0.022
B. Score	8.836	4.061	0.426	−8.187	−5.687	−7.594	−9.079	−5.497
RMSE	0.160	0.108	0.139	0.135	0.181	0.183	0.195	0.178
CP	0.952	0.964	0.957	0.962	0.952	0.955	0.947	0.963

**Table 3:** Simulation results for the complete-case MLE, the MLE, and the pseudo-conditional likelihood method. Here RMSE represents root mean squared error. The results were based on 2,000 runs. There were 2×2×2 = 8 disease subtypes. The model for the intercepts was misspecified. The missingness probabilities depend on the covariate. This is Scenario 1c, where the true values of some of the second-order contrasts of the log-odds ratio parameters were not zero.

Scenario 1c: Additive model (misspecified)					
		θ^(0)^ =0.35	$\theta_{1(2)}^{\left(1\right)}=0.15$	$\theta_{2(2)}^{\left(1\right)}=0$	$\theta_{2(3)}^{\left(1\right)}=0.5$
Bias	CMLE	0.099	−0.248	−0.078	0.096
	MLE	0.100	−0.250	−0.078	0.095
	PCL	0.001	−0.137	0.000	0.103
RMSE	CMLE	0.179	0.287	0.168	0.184
	MLE	0.157	0.277	0.145	0.158
	PCL	0.139	0.188	0.131	0.168

Scenario 1c: Model with second-order contrasts					
		θ^(0)^=0.35	$\theta_{1(2)}^{\left(1\right)}=0.15$	$\theta_{2(2)}^{\left(1\right)}=0$	$\theta_{3(2)}^{\left(1\right)}=0.5$
Bias	CMLE	−0.090	0.023	0.022	0.371
	MLE	−0.080	0.016	0.020	0.363
	PCL	0.015	−0.012	0.004	−0.001
RMSE	CMLE	0.224	0.256	0.250	0.447
	MLE	0.191	0.219	0.217	0.419
	PCL	0.211	0.266	0.272	0.248

**Table 4:** Contingency tables of disease traits configurations among 1; 555 cases from the CPS-II Nutrition Cohort data. Here ER, PR, and NA, stand for estrogen receptor, progesterone receptor, and not available (missing), respectively.

		Histology=Ductal			Histology=Lobular			Histology=Other		
		Stage			Stage			Stage		
		NA	Localized	Distant	NA	Localized	Distant	NA	Localized	Distant
Grade	NA	6	128	38	2	86	32	10	48	10
	Well	2	184	20	0	21	3	0	35	1
	Moderate	10	324	90	0	73	24	0	31	10
	Poor	2	180	104	0	29	13	1	21	17
		PR			PR			PR		
		NA	+	−	NA	+	−	NA	+	−
	NA	323	2	1	66	0	2	70	0	1
ER	+	45	478	110	10	156	39	5	74	12
	−	3	5	121	0	3	7	1	4	17

		ER=NA			ER=+			ER=−		
		Stage			Stage			Stage		
		NA	Localized	Distant	NA	Localized	Distant	NA	Localized	Distant
Grade	NA	16	112	39	2	130	38	0	20	3
	Well	2	46	4	0	188	20	0	6	0
	Moderate	5	112	33	5	288	82	0	28	9
	Poor	2	60	34	1	111	64	0	59	36
		PR			PR			PR		
		NA	+	−	NA	+	−	NA	+	−
	Ductal	323	2	1	45	478	110	3	5	121
Histology	Lobular	66	0	2	10	156	39	0	3	7
	Other	70	0	1	5	74	12	1	4	17

**Table 5:** Results of the CPS-II Nutrition Cohort data analysis with five disease traits and weight gain from age 18 to 1992 as the predictor. Here EST, SE, METH, ER and PR stand for estimate, standard error, method, estrogen receptor and progesterone receptor, respectively.

METH		Ref.	Grade (Well)		Stage (Localized)	Histology		ER Status (ER+)	PR Status (PR+)
			Moderate	Poor	Distant	Lobular	Other	ER−	PR−
		%missing	23.2		21.2	0		30	33.6
		θ^(0)^	$\theta_{1(2)}^{\left(1\right)}$	$\theta_{1(3)}^{\left(1\right)}$	$\theta_{2(2)}^{\left(1\right)}$	$\theta_{3(2)}^{\left(1\right)}$	$\theta_{3(3)}^{\left(1\right)}$	$\theta_{4(2)}^{\left(1\right)}$	$\theta_{5(2)}^{\left(1\right)}$
CMLE	EST	1.312	−0.160	−0.033	0.703	−0.652	0.379	−0.126	−0.879
	SE	0.357	0.386	0.415	0.354	0.443	0.523	0.420	0.339
	*p*-value	<0.001	0.679	0.937	0.047	0.142	0.469	0.764	0.009
MLE	EST	0.961	0.040	0.268	0.795	−0.666	0.404	0.233	−0.693
	SE	0.305	0.332	0.357	0.263	0.307	0.349	0.376	0.308
	*p*-value	0.002	0.904	0.452	0.003	0.030	0.246	0.535	0.025
PCL	EST	1.066	0.025	0.128	0.810	−0.685	0.368	0.883	−1.222
	SE	0.273	0.317	0.346	0.261	0.303	0.351	0.439	0.359
	*p*-value	<0.001	0.937	0.711	0.002	0.024	0.294	0.045	0.001

**Table 6:** Results of the CPS-II Nutrition Cohort data analysis using the PCL method with five disease traits and weight gain was used as the covariate. Here EST, SE, ER and PR stand for estimate, standard error, estrogen receptor and progesterone receptor, respectively. We have included second-order contrasts in the model for the log-odds ratio parameters.

Parameter	θ^(0)^	$\theta_{1(2)}^{\left(1\right)}$	$\theta_{1(3)}^{\left(1\right)}$	$\theta_{2(2)}^{\left(1\right)}$	$\theta_{3(2)}^{\left(1\right)}$	$\theta_{3(3)}^{\left(1\right)}$	$\theta_{4(2)}^{\left(1\right)}$	$\theta_{5(2)}^{\left(1\right)}$	$\theta_{12(2,2)}^{\left(2\right)}$
EST	1.041	0.029	0.157	0.316	−0.502	−0.229	−3.991	−0.118	0.228
SE	0.347	0.440	0.493	0.792	0.807	0.801	1.589	0.617	0.882
*p*-value	0.003	0.948	0.750	0.690	0.534	0.775	0.012	0.849	0.796
Parameter	$\theta_{13(2,2)}^{\left(2\right)}$	$\theta_{13(2,3)}^{\left(2\right)}$	$\theta_{14(2,2)}^{\left(2\right)}$	$\theta_{15(2,2)}^{\left(2\right)}$	$\theta_{12(3,2)}^{\left(2\right)}$	$\theta_{13(3,2)}^{\left(2\right)}$	$\theta_{13(3,3)}^{\left(2\right)}$	$\theta_{14(3,2)}^{\left(2\right)}$	$\theta_{15(3,2)}^{\left(2\right)}$
EST	0.061	−0.290	4.680	−0.840	1.368	−0.112	0.063	3.569	−1.743
SE	0.927	1.228	1.449	1.004	0.904	0.940	1.381	1.298	0.954
*p*-value	0.947	0.813	0.001	0.402	0.130	0.905	0.964	0.006	0.068
Parameter	$\theta_{13(2,2)}^{\left(2\right)}$	$\theta_{13(2,3)}^{\left(2\right)}$	$\theta_{14(2,2)}^{\left(2\right)}$	$\theta_{15(2,2)}^{\left(2\right)}$	$\theta_{12(3,2)}^{\left(2\right)}$	$\theta_{13(3,2)}^{\left(2\right)}$	$\theta_{13(3,3)}^{\left(2\right)}$	$\theta_{14(3,2)}^{\left(2\right)}$	$\theta_{15(3,2)}^{\left(2\right)}$
EST	−0.068	−0.332	0.155	−0.710	1.137	−1.677	1.731	1.124	1.165
SE	0.685	1.132	1.115	0.867	1.240	0.944	1.356	1.027	1.158
*p*-value	0.922	0.770	0.889	0.413	0.359	0.076	0.202	0.274	0.314

## Appendix

### A General Methodology

Suppose that *X* = (*X*_1_, … *X_P_*) is a vector *P* of covariates, *Y* = (*Y*_1_, … *Y_K_*) and carries information on *K* disease traits, and *M* = *M*_1_ × *M*_2_ × … × *M_K_* is the total number of disease subtypes, based on all possible combinations of the various traits. We will use *y* for (*y*_1_, … *y_K_*). Our model is $p_{i,y}\equiv \text{pr}(D_{i}=1,Y_{i}=y|X_{i})=\exp\left(\alpha_{y}+\sum_{p=1}^{P}\beta_{y}^{\left(p\right)}X_{i,p}\right)/\left\{1+\sum_{y}\exp\left(\alpha_{y}+\sum_{p=1}^{P}\beta_{y}^{\left(p\right)}X_{i,p}\right)\right\}$ and $\text{pr}(D_{i}=0|X_{i})=1/\left\{1+\sum_{y}\exp\left(\alpha_{y}+\sum_{p=1}^{P}\beta_{y}^{\left(p\right)}X_{i,p}\right)\right\}$, for *i* = 1, … *n*. For *M* disease subtypes, we have *M* × *P* main regression parameters of interest along with *M* intercept parameters. The log-linear model for the log-odds ratio parameter is

(1) $$\beta_{y}^{\left(p\right)}=\beta_{\left(y_{1},\ldots ,y_{K}\right)}^{\left(p\right)}=\theta^{{\left(0\right)}^{\left(p\right)}}+\sum_{k=1}^{K}{\theta^{\left(1\right)}}_{k(y_{k})}^{\left(p\right)}+\sum_{k=1}^{K}\sum_{k^{\prime }\geq k}^{K}{\theta^{\left(2\right)}}_{kk^{\prime }(y_{k},y_{k^{\prime }})}^{\left(p\right)}+\cdots +{\theta^{\left(K\right)}}_{12\ldots K(y_{1},\ldots ,y_{K})}^{\left(p\right)}.$$

Suppose that *β*^(^*^p^*^)^ is the set of log-odds ratio parameter corresponding to *X_p_*, then the second-stage model can be written as *β*^(^*^p^*^)^ =  *θ*^(^*^p^*^)^. From here on, we denote (*θ*^(1)^*^T^*, … *θ*^(^*^p^*^)^*^T^*)*^T^* by *θ*. For each subject we introduce a vector of binary variables *R* = (*R*_1_, … *R_K_*)*^T^*, where *R_K_* = 1 if the *k^th^* trait is observed and 0 otherwise. For our convenience, we set *R* = (1, … 1)*^T^* for a non-diseased subject. Using our methodology the estimating functions for *θ* are

$$\begin{array}{l} S_{EE,\theta^{\left(p\right)}}\equiv \frac{\partial \;\log\;\left(L_{PCL}\right)}{\partial \theta^{\left(p\right)}}=\sum_{i=1}^{n}D_{i}\sum_{r}I(R_{i}=r)\{X_{i,p}\sum_{y_{i}^{m_{r}}}B_{\left(y_{i}^{o_{r}},y_{i}^{m_{r}}\right)}^{\left(p\right)}\omega_{\left(y_{i}^{o_{r}},y_{i}^{m_{r}}\right)}\\ -\frac{n_{0}^{-1}\sum_{y_{i}^{m_{r}}}\exp(A_{\left(y_{i}^{o_{r}},y_{i}^{m_{r}}\right)}^{T}\xi +\sum_{p=1}^{P}X_{i,p}B_{\left(y_{i}^{o_{r}},y_{i}^{m_{r}}\right)}^{(p)T}\theta^{\left(p\right)})X_{i,p}B_{y_{i}^{o_{r}},y_{i}^{m_{r}}}^{\left(p\right)}+M_{y_{i}^{o_{r}},p}^{\left(1\right)}(Q_{0n})}{n_{0}^{-1}\sum_{y_{i}^{m_{r}}}\exp\left(A_{\left(y_{i}^{o_{r}},y_{i}^{m_{r}}\right)}^{T}\xi +\sum_{p=1}^{P}X_{i,p}B_{\left(y_{i}^{o_{r}},y_{i}^{m_{r}}\right)}^{(p)T}\theta^{\left(p\right)}\right)+M_{y_{i}^{o_{r}},p}^{\left(0\right)}(Q_{0n})}\}, \end{array}$$

where for *k* = 0, 1,

$$\begin{array}{l} M_{y_{i}^{o_{r}},p}^{\left(k\right)}(Q_{0n})=n_{0}^{-1}\sum_{j\in S_{i}/\{i\}}\exp(A_{\left(y_{i}^{o_{r}},y_{i}^{m_{r}}\right)}^{T}\xi +\sum_{p=1}^{P}X_{p}B_{\left(y_{i}^{o_{r}},y_{i}^{m_{r}}\right)}^{(p)T}\theta^{\left(p\right)}){\left(X_{j,p}B_{y_{i}^{o_{r}},y_{i}^{m_{r}}}^{\left(p\right)}\right)}^{⊗k}\\ =\int \sum_{y_{i}^{m_{r}}}\exp\left(A_{\left(y_{i}^{o_{r}},y_{i}^{m_{r}}\right)}^{T}\xi +\sum_{p=1}^{P}X_{p}B_{\left(y_{i}^{o_{r}},y_{i}^{m_{r}}\right)}^{(p)T}\theta^{\left(p\right)}\right){\left(X_{p}B_{\left(y_{i}^{o_{r}},y_{i}^{m_{r}}\right)}^{\left(p\right)}\right)}^{⊗k}{dQ}_{0n}(X), \end{array}$$

and $Q_{0n}(x)=n_{0}^{-1}\sum_{i=1}^{n}I(D_{i}=0,X_{i}=x)$ denotes the empirical distribution function of *X* among the controls which converges in probability to the true distribution of *X* among the controls denoted by *Q*_0_(*x*). Here *a*^⊗^*^k^* = 1, *a*, *aa^T^* for *k* = 0, 1, 2, respectively. We want to clarify that $\sum_{r}\;$ in *S*_E*E*,*θ*(^*p*)^_ signifies a summation over all possible values of the indicator vecto *r*. If there are three traits, then the possible values of *r* are (0, 0, 0), (1, 0, 0), (0, 1, 0), (1, 1, 0), (0, 0, 1), (1, 0, 1), (0, 1, 1) and (1, 1, 1).

The estimating functions for *ξ* are

$$S_{EE,\xi }\equiv \frac{\partial \;\log\;\left(L_{PCL}^{*}\right)}{\partial \xi }=\sum_{i=1}^{n}D_{i}\{\sum_{r}I\left(R_{i}=r\right)\sum_{y_{i}^{m_{r}}}A_{y_{i}^{o_{r}},y_{i}^{m_{r}}}\omega_{y_{i}^{o_{r}},y_{i}^{m_{r}}}-\frac{n_{0}^{-1}\sum_{y}\exp\left(A_{y}^{T}\xi +\sum_{p=1}^{P}X_{i,p}B_{y}^{(p)T}\theta^{\left(p\right)}\right)A_{y}^{T}+N^{\left(1\right)}\left(Q_{0n}\right)}{n_{0}^{-1}\sum_{y}\exp\left(A_{y}^{T}\xi +\sum_{p=1}^{P}X_{i,p}B_{y}^{(p)T}\theta^{\left(p\right)}\right)+N^{\left(0\right)}\left(Q_{0n}\right)}\},$$

where for *k* = 0, 1,

$$\begin{array}{l} N^{\left(k\right)}\left(Q_{0n}\right)=n_{0}^{-1}\sum_{j\in S_{i}/\{i\}}\sum_{y}\exp\left(A_{y}^{T}\xi +\sum_{p=1}^{P}X_{j,p}B_{y}^{(p)T}\theta^{\left(p\right)}\right)A_{y}^{⊗k}\\ =\int \sum_{y}\exp\left(A_{y}^{T}\xi +\sum_{p=1}^{P}X_{p}B_{y}^{(p)T}\theta^{\left(p\right)}\right)A_{y}^{⊗k}{dQ}_{0n}(X). \end{array}$$

We estimate *θ*^(^*^p^*^)^, *p* = 1, … *P*, and *ξ* by solving *S*_EE,*θ*^(*p*)^_ = 0, *p* = 1, … *P*, *S*_EE,_*_ξ_* = 0 simultaneously. Denote the resulting estimator as *η̂* = (*θ̂^T^*, *ξ̂^T^*)*^T^*.

### B Asymptotic Properties

In this section, we discuss the large sample properties of *η̂*. We show that *n*^−1^*S*_E*E*,*θ*^(*p*)^_ → 0 (*p* = 1, … *P*) and *n*^−1^ *S*_E_*_E_*_,_*_ξ_* → 0 in probability, i.e., the estimating equations are asymptotically unbiased.

#### Regularity conditions

Let $S_{n}(\eta )=n^{-1}{\left(S_{\text{EE},\theta^{\left(1\right)}}^{T},\ldots ,S_{\text{EE},\theta^{\left(P\right)}}^{T},S_{\text{EE},\xi }^{T}\right)}^{T}$.

C1 The parameter space for *η* is a compact subset of an Euclidean space.
C2 $0<\exp\left(\sum_{p=1}^{P}X_{p}B_{y}^{\left(p\right)}\theta^{\left(p\right)}\right)<\infty$ for all *θ*^(^*^p^*^)^ and *y*.
C3 0 < exp (  *ξ*) < ∞ for all *ξ* and *y*.
C4 The elements of the second-stage design matrices  and  remain uniformly bounded in absolute value by constants, say  and , respectively.
C5 The information matrix *H_n_* is positive definite.
C6 The deterministic equation *E*{*S_n_*(*η*)} = 0 has only one root in the neighborhood of the true parameters.

Conditions C1–C4 are required for uniform convergence, i.e., $\sup_{\eta }\|S_{n}(\eta )-E\{S_{n}(\eta )\}\|\overset{P}{\to }0$. Condition C5, C6 (identifiability) and the asymptotic unbiasedness of *S_n_*(*η*) for zero (to be proved) together imply convergence of the estimator in probability towards the true value (Theorem 5.9 of [15]).

#### Asymptotic Unbiasedness

Here we first show that $n^{-1}S_{EE,\theta^{\left(p\right)}}\overset{P}{\to }0$ as *n* → ∞ at the true parameter value. Due to the law of large numbers, *n*^−1^*S*_E*E*,*θ*^(*p*)^_ converges to its expectation. In order to calculate this expectation, we shall use the conditional probability that the *i*-th subject has disease of type $y=(y_{i}^{o_{r}},y_{i}^{m_{r}})$ given that there is one diseased subject in the matched set  with this disease type. Hence,

$$\begin{array}{l} E\left(\frac{S_{EE,\theta^{\left(p\right)}}}{n}\right)=E[\sum_{r}\int_{y_{i}^{o_{r}}}\sum_{k\in S_{i}}\frac{\sum_{y_{i}^{m_{r}}}\exp(A_{\left(y_{i}^{o_{r}},y_{i}^{m_{r}}\right)}^{T}\xi +\sum_{p=1}^{P}X_{k,p}B_{\left(y_{i}^{o_{r}},y_{i}^{m_{r}}\right)}^{T}\theta^{\left(p\right)})\pi (r,X_{k})}{\sum_{j\in S_{i}}\sum_{y_{i}^{m_{r}}}\exp(A_{\left(y_{i}^{o_{r}},y_{i}^{m_{r}}\right)}^{T}\xi +\sum_{p=1}^{P}X_{j,p}B_{\left(y_{i}^{o_{r}},y_{i}^{m_{r}}\right)}^{T}\theta^{\left(p\right)})}\\ \times \left\{\frac{\sum_{y_{i}^{m_{r}}}\exp\left(A_{\left(y_{i}^{o_{r}},y_{i}^{m_{r}}\right)}^{T}\xi +\sum_{p=1}^{P}X_{k,p}B_{\left(y_{i}^{o_{r}},y_{i}^{m_{r}}\right)}^{T}\theta^{\left(p\right)}\right)X_{k,p}B_{\left(y_{i}^{o_{r}},y_{i}^{m_{r}}\right)}}{\sum_{y_{i}^{m_{r}}}\exp\left(A_{\left(y_{i}^{o_{r}},y_{i}^{m_{r}}\right)}^{T}\xi +\sum_{p=1}^{P}X_{k,p}B_{\left(y_{i}^{o_{r}},y_{i}^{m_{r}}\right)}^{T}\theta^{\left(p\right)}\right)}-\frac{M_{y_{i}^{o_{r}},p}^{\left(1\right)}\left(Q_{0}\right)}{M_{y_{i}^{o_{r}},p}^{\left(0\right)}\left(Q_{0}\right)}\right\}d\mu \left(y_{i}^{o_{r}}\right)]+o(1). \end{array}$$

Now, the first term on the right hand side above is

$$\begin{array}{l} E[\sum_{r}\int_{y_{i}^{o_{r}}}\sum_{k\in S_{i}}\frac{\sum_{y_{i}^{m_{r}}}\exp\left(A_{\left(y_{i}^{o_{r}},y_{i}^{m_{r}}\right)}^{T}\xi +\sum_{p=1}^{P}X_{k,p}B_{\left(y_{i}^{o_{r}},y_{i}^{m_{r}}\right)}^{T}\theta^{\left(p\right)}\right)\pi (r,X_{k})}{\sum_{j\in S_{i}}\sum_{y_{i}^{m_{r}}}\exp\left(A_{\left(y_{i}^{o_{r}},y_{i}^{m_{r}}\right)}^{T}\xi +\sum_{p=1}^{P}X_{k,p}B_{\left(y_{i}^{o_{r}},y_{i}^{m_{r}}\right)}^{T}\theta^{\left(p\right)}\right)}\\ \times \frac{\sum_{y_{i}^{m_{r}}}\exp\left(A_{\left(y_{i}^{o_{r}},y_{i}^{m_{r}}\right)}^{T}\xi +\sum_{p=1}^{P}X_{k,p}B_{\left(y_{i}^{o_{r}},y_{i}^{m_{r}}\right)}^{T}\theta^{\left(p\right)}X\right)X_{k,p}B_{\left(y_{i}^{o_{r}},y_{i}^{m_{r}}\right)}}{\sum_{y_{i}^{m_{r}}}\exp\left(A_{\left(y_{i}^{o_{r}},y_{i}^{m_{r}}\right)}^{T}\xi +\sum_{p=1}^{P}B_{\left(y_{i}^{o_{r}},y_{i}^{m_{r}}\right)}^{T}\theta^{\left(p\right)}\right)}d\mu \left(y_{i}^{o_{r}}\right)]\\ =E\left[\sum_{r}\int_{y_{i}^{o_{r}}}\frac{\sum_{k\in S_{i}}\sum_{y_{i}^{m_{r}}}\exp(A_{\left(y_{i}^{o_{r}},y_{i}^{m_{r}}\right)}^{T}\xi +\sum_{p=1}^{P}X_{k,p}B_{\left(y_{i}^{o_{r}},y_{i}^{m_{r}}\right)}^{T}\theta^{\left(p\right)})X_{k,p}B_{\left(y_{i}^{o_{r}},y_{i}^{m_{r}}\right)}\pi (r,X_{k})}{\sum_{k\in S_{i}}\sum_{y_{i}^{m_{r}}}\exp(A_{\left(y_{i}^{o_{r}},y_{i}^{m_{r}}\right)}^{T}\xi +\sum_{p=1}^{P}X_{j,p}B_{\left(y_{i}^{o_{r}},y_{i}^{m_{r}}\right)}^{T}\theta^{\left(p\right)})}d\mu \left(y_{i}^{o_{r}}\right)\right], \end{array}$$

and the second term is

$$=E\left[\sum_{r}\int_{y_{i}^{o_{r}}}\frac{\sum_{k\in S_{i}}\sum_{y_{i}^{m_{r}}}\exp\left(A_{\left(y_{i}^{o_{r}},y_{i}^{m_{r}}\right)}^{T}\xi +\sum_{p=1}^{P}X_{k,p}B_{\left(y_{i}^{o_{r}},y_{i}^{m_{r}}\right)}^{T}\theta^{\left(p\right)}\right)\pi (r,X_{k})}{\sum_{k\in S_{i}}\sum_{y_{i}^{m_{r}}}\exp\left(A_{\left(y_{i}^{o_{r}},y_{i}^{m_{r}}\right)}^{T}\xi +\sum_{p=1}^{P}X_{j,p}B_{\left(y_{i}^{o_{r}},y_{i}^{m_{r}}\right)}^{T}\theta^{\left(p\right)}\right)}\times \frac{M_{y_{i}^{o_{r}},p}^{\left(1\right)}(Q_{0})}{M_{y_{i}^{o_{r}},p}^{\left(0\right)}(Q_{0})}d\mu \left(y_{i}^{o_{r}}\right)\right].$$

The difference between the two terms is easily seen to be asymptotically the expected weighted conditional covariance between *π*(*r*, *X*) and $X_{\cdot ,p}B_{\left(y_{i}^{o_{r}},y_{i}^{m_{r}}\right)}$ with weight

$$\frac{\exp\left(A_{\left(y_{i}^{o_{r}},y_{i}^{m_{r}}\right)}^{T}\xi +\sum_{p=1}^{P}X_{j,p}B_{\left(y_{i}^{o_{r}},y_{i}^{m_{r}}\right)}^{T}\theta^{\left(p\right)}\right)}{\sum_{k\in S_{i}}\sum_{y_{i}^{m_{r}}}\exp(A_{\left(y_{i}^{o_{r}},y_{i}^{m_{r}}\right)}^{T}\xi +\sum_{p=1}^{P}X_{j,p}B_{\left(y_{i}^{o_{r}},y_{i}^{m_{r}}\right)}^{T}\theta^{\left(p\right)})}.$$

Let

$$\begin{array}{l} {\mathrm{cov}}^{w}\left\{\pi (r,X),X_{\cdot ,p}B_{\left(y_{i}^{o_{r}},y_{i}^{m_{r}}\right)}\right\}\\ =\sum_{j\in S_{i}}\sum_{y_{i}^{m_{r}}}\frac{\exp\left(A_{\left(y_{i}^{o_{r}},y_{i}^{m_{r}}\right)}^{T}\xi +\sum_{p=1}^{P}X_{j,p}B_{\left(y_{i}^{o_{r}},y_{i}^{m_{r}}\right)}^{T}\theta^{\left(p\right)}\right)}{\sum_{j\in S_{i}}\sum_{y_{i}^{m_{r}}}\exp(A_{\left(y_{i}^{o_{r}},y_{i}^{m_{r}}\right)}^{T}\xi +\sum_{p=1}^{P}X_{j,p}B_{\left(y_{i}^{o_{r}},y_{i}^{m_{r}}\right)}^{T}\theta^{\left(p\right)})}\left\{\pi \left(r,X_{j}\right)-\bar{\pi }(r,X)\right\}\\ \times \left(X_{j,p}B_{\left(y_{i}^{o_{r}},y_{i}^{m_{r}}\right)}-\frac{M_{y_{i}^{o_{r}},p}^{\left(1\right)}\left(Q_{0}\right)}{M_{y_{i}^{o_{r}},p}^{\left(0\right)}\left(Q_{0}\right)}\right), \end{array}$$

where

$$\bar{\pi }(r,X)=\sum_{j\in S_{i}}\sum_{y_{i}^{m_{r}}}\frac{\exp\left(A_{\left(y_{i}^{o_{r}},y_{i}^{m_{r}}\right)}^{T}\xi +\sum_{p=1}^{P}X_{j,p}B_{\left(y_{i}^{o_{r}},y_{i}^{m_{r}}\right)}^{T}\theta^{\left(p\right)}\right)\pi \left(r,X_{j}\right)}{\sum_{j\in S_{i}}\sum_{y_{i}^{m_{r}}}\exp\left(A_{\left(y_{i}^{o_{r}},y_{i}^{m_{r}}\right)}^{T}\xi +\sum_{p=1}^{P}X_{j,p}B_{\left(y_{i}^{o_{r}},y_{i}^{m_{r}}\right)}^{T}\theta^{\left(p\right)}\right)}$$

Then, we can write

$$E\left(\frac{S_{EE,\theta^{\left(p\right)}}}{n}\right)=E\left[\sum_{r}\int_{y_{i}^{o_{r}}}c{ov}^{w}\left\{\pi (r,X),X_{\cdot ,p}B_{\left(y_{i}^{o_{r}},y_{i}^{m_{r}}\right)}\right\}d\mu \left(y_{i}^{o_{r}}\right)\right]+o(1)=o(1),$$

where the last equality follows due to the fact that Σ*_r_π*(*r*, *X*) = 1.

Similarly, due to the law of large numbers, *n*^−1^*S*_E_*_E_*_,_*_ξ_* converges to its expectation. In order to calculate this expectation, we shall use the conditional probability that the *i*-th subject has disease of type $y=(y_{i}^{o_{r}},y_{i}^{m_{r}})$ given that there is one diseased subject in the matched set  but without specifying any disease subtype information. Hence,

$$\begin{array}{l} E\left(\frac{S_{EE,\xi }}{n}\right)=E[\int_{y_{i}^{o_{r}}}\sum_{r}\sum_{k\in S_{i}}\left(r,X_{k}\right)\frac{\sum_{y_{i}^{m_{r}}}\exp\left(A_{\left(y_{i}^{o_{r}},y_{i}^{m_{r}}\right)}^{T}\xi +\sum_{p=1}^{P}X_{k,p}B_{\left(y_{i}^{o_{r}},y_{i}^{m_{r}}\right)}^{T}\theta^{\left(p\right)}\right)}{\sum_{j\in S_{i}}\sum_{y}\exp\left(A_{y}^{T}\xi +\sum_{p=1}^{P}X_{j,p}B_{y}^{T}\theta^{\left(p\right)}\right)}\\ \times \left\{\frac{\sum_{y_{i}^{m_{r}}}\exp\left(A_{\left(y_{i}^{o_{r}},y_{i}^{m_{r}}\right)}^{T}\xi +\sum_{p=1}^{P}X_{k,p}B_{\left(y_{i}^{o_{r}},y_{i}^{m_{r}}\right)}^{T}\theta^{\left(p\right)}\right)A_{\left(y_{i}^{o_{r}},y_{i}^{m_{r}}\right)}}{\sum_{y_{i}^{m_{r}}}\exp\left(A_{\left(y_{i}^{o_{r}}y_{i}^{m_{r}}\right)}^{T}\xi +\sum_{P=1}^{P}X_{k,p}B_{\left(y_{i}^{o_{r}},y_{i}^{m_{r}}\right)}^{T}\theta^{\left(p\right)}\right)}-\frac{N^{\left(1\right)}\left(Q_{0}\right)}{N^{\left(0\right)}\left(Q_{0}\right)}\right\}d\mu \left(y_{i}^{o_{r}}\right)]+o(1)\\ =E[\frac{\sum_{r}\sum_{k\in S_{i}}\left(r,X_{k}\right)\int_{y_{i}^{o_{r}}}\sum_{y_{i}^{m_{r}}}\exp\left(A_{\left(y_{i}^{o_{r}},y_{i}^{m_{r}}\right)}^{T}\xi +\sum_{p=1}^{P}X_{k,p}B_{\left(y_{i}^{o_{r}},y_{i}^{m_{r}}\right)}^{T}\theta^{\left(p\right)}\right)A_{\left(y_{i}^{o_{r}},y_{i}^{m_{r}}\right)}d\mu \left(y_{i}^{o_{r}}\right)}{\sum_{j\in S_{i}}\sum_{y}\exp\left(A_{y}^{T}\xi +\sum_{p=1}^{P}X_{j,p}B_{y}^{T}\theta^{\left(p\right)}\right)}\\ -\frac{\sum_{r}\sum_{k\in S_{i}}\left(r,X_{k}\right)=\int_{y_{i}^{o_{r}}}\sum_{y_{i}^{m_{r}}}\exp\left(A_{\left(y_{i}^{o_{r}},y_{i}^{m_{r}}\right)}^{T}\xi +\sum_{p=1}^{P}X_{k,p}B_{\left(y_{i}^{o_{r}},y_{i}^{m_{r}}\right)}^{T}\theta^{\left(p\right)}\right)d\mu \left(y_{i}^{o_{r}}\right)}{\sum_{j\in S_{i}}\sum_{y}\exp\left(A_{y}^{T}\xi +\sum_{p=1}^{P}X_{j,p}B_{y}^{T}\theta^{\left(p\right)}\right)}\times \frac{N^{\left(1\right)}(Q_{0})}{N^{\left(0\right)}(Q_{0})}]+o(1)\\ =E[\frac{\sum_{r}\sum_{k\in S_{i}}\pi \left(r,X_{k}\right)\sum_{y}\exp\left(A_{y}^{T}\xi +\sum_{p=1}^{P}X_{k,p}B_{y}^{T}\theta^{\left(p\right)}\right)A_{y}}{\sum_{j\in S_{i}}\sum_{y}\exp\left(A_{y}^{T}\xi +\sum_{p=1}^{P}X_{j,p}B_{y}^{T}\theta^{\left(p\right)}\right)}\\ -\frac{\sum_{r}\sum_{k\in S_{i}}\pi \left(r,X_{k}\right)\sum_{y}\exp\left(A_{y}^{T}\xi +\sum_{p=1}^{P}X_{k,p}B_{y}^{T}\theta^{\left(p\right)}\right)}{\sum_{j\in S_{i}}\sum_{y}\exp\left(A_{y}^{T}\xi +\sum_{p=1}^{P}X_{j,p}B_{y}^{T}\theta^{\left(p\right)}\right)}\times \frac{N^{\left(1\right)}\left(Q_{0}\right)}{N^{\left(0\right)}\left(Q_{0}\right)}]+o(1). \end{array}$$

Now using the facts that Σ*_r_π*(*r*, *X_k_*) = 1 and

$$\frac{\sum_{k\in S_{i}}\sum_{y}\exp\left(A_{y}^{T}\xi +\sum_{p=1}^{P}X_{k,p}B_{y}^{T}\theta^{\left(p\right)}\right)A_{y}}{\sum_{j\in S_{i}}\sum_{y}\exp\left(A_{y}^{T}\xi +\sum_{p=1}^{P}X_{j,p}B_{y}^{T}\theta^{\left(p\right)}\right)}\overset{P}{\to }\frac{N^{\left(1\right)}\left(Q_{0}\right)}{N^{\left(0\right)}\left(Q_{0}\right)},$$

we obtain that $n^{-1}S_{EE,\xi }\overset{P}{\to }0$.

#### Asymptotic Normality

Note that for large *n*

(B1) $$\begin{array}{l} \frac{1}{\sqrt{n}}S_{EE,\theta^{\left(p\right)}}=\frac{1}{\sqrt{n}}\sum_{i=1}^{n}D_{i}\sum_{r}I\left(R_{i}=r\right)\left\{X_{i,p}\sum_{y_{i}^{m_{r}}}B_{\left(y_{i}^{o_{r}},y_{i}^{m_{r}}\right)}\omega_{\left(y_{i}^{o_{r}},y_{i}^{m_{r}},X_{i}\right)}-\frac{M_{y_{i}^{o_{r}},p}^{\left(1\right)}\left(Q_{0n}\right)}{M_{y_{i}^{o_{r}},p}^{\left(0\right)}\left(Q_{0n}\right)}\right\}+o(1)\\ =\frac{1}{\sqrt{n}}\sum_{i=1}^{n}D_{i}\sum_{r}I\left(R_{i}=r\right)\left\{X_{i,p}\sum_{y_{i}^{m_{r}}}B_{\left(y_{i}^{o_{r}},y_{i}^{m_{r}}\right)}\omega_{\left(y_{i}^{o_{r}},y_{i}^{m_{r}},X_{i}\right)}-\frac{M_{y_{i}^{o_{r}},p}^{\left(1\right)}\left(Q_{0}\right)}{M_{y_{i}^{o_{r}},p}^{\left(0\right)}\left(Q_{0}\right)}\right\}\\ -\frac{1}{\sqrt{n}}\sum_{i=1}^{n}D_{i}\sum_{r}I\left(R_{i}=r\right)\left\{\frac{M_{y_{i}^{o_{r}},p}^{\left(1\right)}\left(Q_{0n}\right)}{M_{y_{i}^{o_{r}},p}^{\left(0\right)}\left(Q_{0n}\right)}-\frac{M_{y_{i}^{o_{r}},p}^{\left(1\right)}\left(Q_{0}\right)}{M_{y_{i}^{o_{r}},p}^{\left(0\right)}\left(Q_{0}\right)}\right\}+o_{p}(1). \end{array}$$

Let ${\hat{a}}_{i}=M_{y_{i}^{o_{r}},p}^{\left(1\right)}\left(Q_{0n}\right)$ and ${\hat{b}}_{i}=M_{y_{i}^{o_{r}},p}^{\left(0\right)}\left(Q_{0n}\right)$. Then using the fact that

$$\frac{{\hat{a}}_{i}}{{\hat{b}}_{i}}-\frac{a_{i}}{b_{i}}=\frac{{\hat{a}}_{i}-a_{i}}{b_{i}}-\frac{a_{i}}{b_{i}^{2}}\left({\hat{b}}_{i}-b_{i}\right)+o_{p}\left(n^{-1/2}\right),$$

the summand of the second term of (B1) is

(B2) $$\begin{array}{l} \frac{M_{y_{i}^{o_{r}},p}^{\left(1\right)}\left(Q_{0n}\right)}{M_{y_{i}^{o_{r}},p}^{\left(0\right)}\left(Q_{0n}\right)}-\frac{M_{y_{i}^{o_{r}},p}^{\left(1\right)}\left(Q_{0}\right)}{M_{y_{i}^{o_{r}},p}^{\left(0\right)}\left(Q_{0}\right)}=\frac{1}{nM_{y_{i}^{o_{r}},p}^{\left(0\right)}\left(Q_{0}\right)}\sum_{j=1}^{n}\left(1-D_{j}\right)\sum_{y_{i}^{m_{r}}}\exp\left(A_{\left(y_{i}^{o_{r}},y_{i}^{m_{r}}\right)}^{T}\xi +\sum_{p=1}^{P}X_{j,p}B_{\left(y_{i}^{o_{r}},y_{i}^{m_{r}}\right)}^{T}\theta^{\left(p\right)}\right)\\ \times \left\{X_{j,p}B_{\left(y_{i}^{o_{r}},y_{i}^{m_{r}}\right)}-\frac{M_{y_{i}^{o_{r}},p}^{\left(1\right)}\left(Q_{0}\right)}{M_{y_{i}^{o_{r}},p}^{\left(0\right)}\left(Q_{0}\right)}\right\}+o_{p}\left(n^{-1/2}\right). \end{array}$$

Plugging (B2) into (B1) and changing the order of the two summations in the second term, we have

$$\begin{array}{l} \frac{1}{\sqrt{n}}S_{EE,\theta^{\left(p\right)}}=\frac{1}{\sqrt{n}}\sum_{i=1}^{n}\sum_{r}I\left(R_{i}=r\right)D_{i}\left\{X_{i,p}\sum_{y_{i}^{m_{r}}}B_{\left(y_{i}^{o_{r}},y_{i}^{m_{r}}\right)}\omega_{\left(y_{i}^{o_{r}},y_{i}^{m_{r}},X_{i}\right)}-\frac{M_{y_{i}^{o_{r}},p}^{\left(1\right)}\left(Q_{0}\right)}{M_{y_{i}^{o_{r}},p}^{\left(0\right)}\left(Q_{0}\right)}\right\}\\ -\frac{1}{\sqrt{n}}\sum_{i=1}^{n}(1-D_{i})\frac{1}{n}\sum_{j=1}^{n}\sum_{r}\left(R_{j}=r\right)D_{j}\frac{1}{M_{y_{j}^{o_{r}},p}^{\left(0\right)}\left(Q_{0}\right)}\sum_{y_{j}^{m_{r}}}\exp(A_{\left(y_{j}^{o_{r}},y_{j}^{m_{r}}\right)}^{T}\xi \\ +\sum_{p=1}^{P}X_{i,p_{i,p}}B_{\left(y_{j}^{o_{r}},y_{j}^{m_{r}}\right)}^{T}\theta^{\left(p\right)})\left\{X_{i,p}B_{\left(y_{j}^{o_{r}},y_{j}^{m_{r}}\right)}-\frac{M_{y_{j}^{o_{r}},p}^{\left(1\right)}\left(Q_{0}\right)}{M_{y_{j}^{o_{r}},p}^{\left(0\right)}\left(Q_{0}\right)}\right\}+o_{p}(1). \end{array}$$

Finally, applying the strong law of large numbers and the Slutsky’s Theorem, we obtain $n^{-1/2}S_{\text{EE},\theta^{\left(p\right)}}\overset{d}{=}n^{-1/2}\sum_{i=1}^{n}\Phi_{i,\theta^{\left(p\right)}}(\theta ,\xi )$ asymptotically. Similarly, for large *n*,

(B3) $$\begin{array}{l} \frac{1}{\sqrt{n}}S_{EE,\xi }=\frac{1}{\sqrt{n}}\sum_{i=1}^{n}\sum_{r}\left(R_{i}=r\right)D_{i}\left\{\sum_{y_{i}^{m_{r}}}\omega_{\left(y_{i}^{o_{r}},y_{i}^{m_{r}},X_{i}\right)}A_{\left(y_{i}^{o_{r}},y_{i}^{m_{r}}\right)}-\frac{N^{\left(1\right)}\left(Q_{0n}\right)}{N^{\left(0\right)}\left(Q_{0n}\right)}\right\}+o(1)\\ =\frac{1}{\sqrt{n}}\sum_{i=1}^{n}\sum_{r}I\left(R_{i}=r\right)D_{i}\left\{\sum_{y_{i}^{m_{r}}}\omega_{\left(y_{i}^{o_{r}},y_{i}^{m_{r}},X_{i}\right)}A_{\left(y_{i}^{o_{r}},y_{i}^{m_{r}}\right)}-\frac{N^{\left(1\right)}\left(Q_{0}\right)}{N^{\left(0\right)}\left(Q_{0}\right)}\right\}-\frac{1}{\sqrt{n}}\sum_{i=1}^{n}\sum_{r}I\left(R_{i}=r\right)D_{i}\left\{\frac{N^{\left(1\right)}\left(Q_{0n}\right)}{N^{\left(0\right)}\left(Q_{0n}\right)}-\frac{N^{\left(1\right)}\left(Q_{0}\right)}{N^{\left(0\right)}\left(Q_{0}\right)}\right\}+o_{p}(1) \end{array}$$

Employing the same technique as that used in (B2), we can write

(B4) $$\frac{N^{\left(1\right)}\left(Q_{0n}\right)}{N^{\left(0\right)}\left(Q_{0n}\right)}-\frac{N^{\left(1\right)}\left(Q_{0}\right)}{N^{\left(0\right)}\left(Q_{0}\right)}=\frac{1}{nN^{\left(0\right)}\left(Q_{0}\right)}\sum_{j=1}^{n}\left(1-D_{j}\right)\sum_{y}\exp\left(A_{y}^{T}\xi +\sum_{p=1}^{P}X_{j,p}B_{y}^{T}\theta^{\left(p\right)}\right)\times \left\{A_{y}-\frac{N^{\left(1\right)}\left(Q_{0}\right)}{N^{\left(0\right)}\left(Q_{0}\right)}\right\}+o_{p}\left(n^{-1/2}\right).$$

Plugging (B4) into (B3) and changing the order of the two summations in the second term, we have

$$\begin{array}{l} \frac{1}{\sqrt{n}}S_{EE,\xi }=\frac{1}{\sqrt{n}}\sum_{i=1}^{n}\sum_{r}I\left(R_{i}=r\right)D_{i}\left\{\sum_{y_{i}^{m_{r}}}\omega_{\left(y_{i}^{o_{r}},y_{i}^{m_{r}},X_{i}\right)}A_{\left(y_{i}^{o_{r}},y_{i}^{m_{r}}\right)}-\frac{N^{\left(1\right)}\left(Q_{0n}\right)}{N^{\left(1\right)}\left(Q_{0n}\right)}\right\}-\frac{1}{\sqrt{n}}\sum_{i=1}^{n}\left(1-D_{i}\right)\\ \times \sum_{j=1}^{n}\sum_{r}I\left(R_{j}=r\right)D_{j}\frac{1}{nN^{\left(0\right)}\left(Q_{0}\right)}\sum_{y}\exp\left(A_{y}^{T}\xi +\sum_{p=1}^{p}X_{i,p}B_{y}^{T}\theta^{\left(p\right)}\right)\left\{A_{y}-\frac{N^{\left(1\right)}\left(Q_{0}\right)}{N^{\left(0\right)}\left(Q_{0}\right)}\right\}+o_{p}(1)\\ \overset{d}{=}\frac{1}{\sqrt{n}}\sum_{i=1}^{n}\sum_{r}I\left(R_{i}=r\right)D_{i}\left\{\sum_{y_{i}^{m_{r}}}\omega_{\left(y_{i}^{o_{r}},y_{i}^{m_{r}},X_{i}\right)}A_{\left(y_{i}^{o_{r}},y_{i}^{m_{r}}\right)}-\frac{N^{\left(1\right)}\left(Q_{0n}\right)}{N^{\left(1\right)}\left(Q_{0n}\right)}\right\}-\frac{1}{\sqrt{n}}\sum_{i=1}^{n}\left(1-D_{i}\right)\\ \times E\left[\sum_{r}I\left(R_{j}=r\right)D_{1}\frac{1}{N^{\left(0\right)}\left(Q_{0}\right)}\sum_{y}\exp\left(A_{y}^{T}\xi +\sum_{p=1}^{p}X_{i,p}B_{y}^{T}\theta^{\left(p\right)}\right)\left\{A_{y}-\frac{N^{\left(1\right)}\left(Q_{0}\right)}{N^{\left(0\right)}\left(Q_{0}\right)}\right\}\right]+o_{p}(1). \end{array}$$

The last equality follows due to the application of the strong law of large numbers and Slutsky’s Theorem. Thus we have shown that *S*_EE,_*_θ_* and *S*_EE,_*_ξ_* are approximately a sum of asymptotically independent random variables whose means are zero. Now,

$$\sqrt{n}\left[\begin{array}{c} \hat{\theta }-\theta \\ \hat{\xi }-\xi \end{array}\right]=H_{n}^{-1}\frac{1}{\sqrt{n}}S_{n}(\eta )+o_{p}(1)+H^{-1}\frac{1}{\sqrt{n}}\sum_{i=1}^{n}\left[\begin{array}{c} \Phi_{i,\theta }(\theta ,\xi )\\ \Phi_{i,\xi }(\theta ,\xi ) \end{array}\right]+o_{p}(1),$$

where *θ* = (*θ*^(1)^*T*^^, ··· *θ*^(*p*)^*T*^)*T*^,

$$H=\left[\begin{array}{lllll} H_{11} & H_{12} & \cdots & H_{1P} & H_{1\xi }\\ \vdots & \; & \; & \; & \;\\ H_{P1} & H_{P2} & \cdots & H_{PP} & H_{P\xi }\\ H_{\xi 1} & H_{\xi 2} & \cdots & H_{\xi P} & H_{\xi \xi } \end{array}\right]=\lim_{n\to \infty }H_{n},\;H_{n}\equiv -\frac{1}{n}\left[\begin{array}{cc} \frac{\partial S_{EE,\theta }}{\partial \theta } & \frac{\partial S_{EE,\theta }}{\partial \xi }\\ \frac{\partial S_{EE,\xi }}{\partial \theta } & \frac{\partial S_{EE,\xi }}{\partial \xi } \end{array}\right],$$

and the asymptotically independent terms are $\Phi_{i,\theta }(\theta ,\xi )={\left(\Phi_{i,\theta^{\left(1\right)}}^{T}(\theta ,\xi ),\ldots ,\Phi_{i,\theta^{\left(P\right)}}^{T}(\theta ,\xi ),\Phi_{i,\xi }^{T}(\theta ,\xi )\right)}^{T}$. Also,

$$\begin{array}{l} \Phi_{i,\theta^{\left(p\right)}}(\theta ,\xi )=\sum_{r}I\left(R_{i}=r\right)D_{i}\left\{\sum_{y_{i}^{m_{r}}}\omega_{\left(y_{i}^{o_{r}},y_{i}^{m_{r}},X_{i}\right)}X_{i,p}B_{\left(y_{i}^{o_{r}},y_{i}^{m_{r}}\right)}-\frac{M_{y^{o_{r}},p}^{\left(1\right)}\left(Q_{0}\right)}{M_{y^{o_{r}},p}^{\left(0\right)}\left(Q_{0}\right)}\right\}\\ -\left(1-D_{i}\right)E[\sum_{r}I(R=r)\times \frac{D}{M_{y^{o_{r}}}^{\left(0\right)}\left(Q_{0}\right)}\sum_{y^{m_{r}}}\exp(A_{\left(y^{o_{r}},y^{m_{r}}\right)}^{T}\xi \\ +\sum_{p=1}^{p}X_{i,p}B_{\left(y^{o_{r}},y^{m_{r}}\right)}^{T}\theta^{\left(p\right)})\left\{X_{i,p}B_{\left(y^{o_{r}},y^{m_{r}}\right)}-\frac{M_{y^{o_{r}},p}^{\left(1\right)}\left(Q_{0}\right)}{M_{y^{o_{r}},p}^{\left(0\right)}\left(Q_{0}\right)}\right\}|X_{i}],\\ \Phi_{i,\xi }(\theta ,\xi )=\sum_{r}I\left(R_{i}=r\right)D_{i}\left\{\sum_{y_{i}^{m_{r}}}\omega_{\left(y_{i}^{o_{r}},y_{i}^{m_{r}},X_{i}\right)}A_{\left(y_{i}^{o_{r}},y_{i}^{m_{r}}\right)}-\frac{N^{\left(1\right)}\left(Q_{0}\right)}{N^{\left(0\right)}\left(Q_{0}\right)}\right\}\\ -\left(1-D_{i}\right)E\left[\sum_{r}I(R=r)\frac{D}{N^{\left(0\right)}\left(Q_{0}\right)}\sum_{y}\exp\left(A_{y}^{T}\xi +\sum_{p=1}^{P}X_{i,p}B_{y}^{T}\theta^{\left(p\right)}\right)\left\{A_{y}-\frac{N^{\left(1\right)}\left(Q_{0}\right)}{N^{\left(0\right)}\left(Q_{0}\right)}\right\}|X_{i}\right], \end{array}$$

where *Q*_0_ represents the true distribution of *X* among the controls.

Therefore, the asymptotic covariance of *η̂* can be consistently estimated by

$$H_{n}^{-1}\sum_{i=1}^{n}{\left[\begin{array}{c} {\hat{\Phi }}_{i,\theta }(\hat{\theta },\hat{\xi })\\ {\hat{\Phi }}_{i,\xi }(\hat{\theta },\hat{\xi }) \end{array}\right]}^{⊗2}H_{n}^{-T},$$

where ${\hat{\Phi }}_{i,\theta }^{T}(\hat{\theta },\hat{\xi })$ and ${\hat{\Phi }}_{i,\xi }^{T}(\hat{\theta },\hat{\xi })$ are obtained by replacing the expectations by the empirical averages, *Q*_0_ by *Q*_0_*_n_*, and the true parameters by their consistent estimators.
